# Detection, Reconstruction, and Overheating Warning of Three-Dimensional Dynamic Temperature Fields Inside Conductive Polymer Gels

**DOI:** 10.3390/polym18172141

**Published:** 2026-09-02

**Authors:** Jiachen Zhang, Kaixuan Ni, Xiangfu Wang

**Affiliations:** College of Electronic and Optical Engineering and College of Flexible Electronics (Future Technology), Nanjing University of Posts and Telecommunications, Nanjing 210023, China; b25020218@njupt.edu.cn (J.Z.); b23021724@njupt.edu.cn (K.N.)

**Keywords:** three-dimensional temperature field inversion, focused light-field infrared imaging, inverse heat conduction problem, causal online reconstruction, ETP-Causal LSQR

## Abstract

Conductive polymer gel materials are inherently susceptible to localized overheating during electrical heating owing to spatially nonuniform conductivity distributions, while their internal three-dimensional temperature fields remain challenging to monitor in real time through noncontact means. Traditional inversion methods, such as Tikhonov-LSQR and TV-ADMM, perform frame-by-frame spatial regularization. The former enforces global smoothness, while the latter preserves sharp edges—but neither exploits the temporal evolution of the temperature field governed by the heat conduction equation, leading to unstable reconstructions in deep regions. To address this limitation, we propose a comprehensive methodology for the calibration, reconstruction, and overheating warning of three-dimensional dynamic temperature fields based on the focused light-field infrared camera. A forward electro-thermal coupled heat conduction model is established to characterize the transient temperature evolution within the gel throughout the heating process. Concurrently, a forward imaging model and its corresponding linear system matrix are constructed for focused light-field infrared imaging, enabling the acquisition of infrared light-field images and subsequent reconstruction of the three-dimensional dynamic temperature field. Furthermore, we develop an ETP-Causal LSQR online inversion algorithm tailored for real-time overheating warning during gel heating. Unlike conventional spatial regularization methods, we incorporate a temporal physical prior: the previous reconstruction is propagated through the heat conduction equation to predict the current temperature field, and this prediction is introduced as a soft constraint into the LSQR solver. The algorithm strictly respects causality, using only current measurements and historical reconstructions. Comparative results demonstrate that the proposed method consistently outperforms conventional Tikhonov-LSQR and TV-ADMM algorithms across multiple aspects, including reconstruction accuracy, noise robustness, physical consistency, cross-operating condition generalization, and computational efficiency, thereby validating the effectiveness and broad applicability of the physically constrained causal inversion framework.

## 1. Introduction

Flexible electronics have undergone rapid development in recent years and have gained increasingly widespread applications in wearable devices, flexible displays, electronic skins, and biomedical engineering. As core components of thermal management systems, conductive polymer-based flexible heating devices play a key role in wearable thermotherapy, flexible heaters, and skin-attachable thermal management systems. PEDOT:PSS (poly(3,4-ethylenedioxythiophene):polystyrene sulfonate), as an intrinsically conductive polymer composed of a flexible substrate, a conductive polymer gel heating layer and terminal electrodes, has emerged as one of the most representative materials in flexible heating devices by virtue of its excellent solution processability, favorable mechanical flexibility and tunable electrical conductivity [1,2,3]. The electrical conductivity of PEDOT:PSS originates from delocalized polarons and bipolarons on the conjugated PEDOT backbone [4,5]. Upon oxidation, positively charged hole carriers are generated on the PEDOT segments and are transported along the conjugated backbone via intrachain hopping and interchain tunneling [5,6]. The insulating PSS phase serves as a charge balancing anionic matrix, and its phase-separated morphology and solvent treatment strongly affect the formation of conductive pathways and carrier mobility [4,6,7]. Under an applied electric field, the Joule heating power density is given by $J\cdot E=\sigma |E{|}^{2}$. Therefore, spatial nonuniformity in the electrical conductivity directly leads to a nonuniform Joule heat source [8,9]. Meanwhile, the conductivity is itself temperature-dependent, and its increase in high-temperature regions may further amplify local Joule heating through positive feedback [7,8,10]. This electro-thermal coupling is particularly pronounced in conductive polymers, so that variations in electrode contact resistance and microstructural inhomogeneities can both be converted into localized heat-source enhancement [6,9].

Studies have demonstrated that PEDOT:PSS-based composite flexible heaters can achieve rapid thermal response at low driving voltages [11,12,13]. However, these devices face prominent safety hazards in practical applications: spatially nonuniform electrical conductivity distributions, variations in electrode contact resistance and nonuniform distributions of Joule heat sources caused by localized material thickness fluctuations. Such nonuniform thermal effects give rise to localized high-temperature core zones within the device—namely, three-dimensional hot spots surrounded by regions of lower temperature. Given that these devices are typically in direct contact with or tightly attached to human skin, failure to identify these localized high-temperature core zones in a timely manner may lead to uneven heating, local scalding, and even overheating risks in skin-contact areas. Therefore, achieving real-time monitoring, reconstruction, and overheating warning of the internal three-dimensional temperature field of flexible heating patches without compromising the structural integrity of the devices constitutes a critical challenge urgently demanding resolution in the field of flexible electronic thermal management.

Light-field imaging technology, as a non-intrusive temperature measurement approach, offers a promising pathway for the acquisition of three-dimensional temperature field. Light-field infrared camera, by integrating a microlens array between the main lens and the detector, enables simultaneous capture of both spatial position and propagation direction information of incident light rays in a single exposure—namely four-dimensional light-field data [14,15]. By acquiring four-dimensional light-field information, light-field infrared cameras extend the dimensionality of conventional two-dimensional infrared imaging, rendering it possible to reconstruct internal three-dimensional temperature field distributions from multi-view infrared observations. In recent years, numerous scholars have conducted in-depth research in the field of temperature field reconstruction based on light-field imaging. For instance, a geometric calibration method for focused light-field cameras was proposed by Sun et al. and subsequently applied to reconstruct three-dimensional flame temperature fields [16]. Recent advances in light-field imaging for flow-field and temperature measurements were systematically reviewed by Niu et al. [17]. In subsequent work, Shi et al. established a focused light-field convolution imaging model and successfully reconstructed the three-dimensional temperature distribution of diffusion flames [18]. Drawing on light-field tomography theory, Niu et al. developed a method for reconstructing three-dimensional luminous flame temperature fields with a standard plenoptic camera. By performing three-dimensional deconvolution using the frequency-domain nearest neighbor method, they recovered the temperature distributions of both axisymmetric and non-axisymmetric flames [19]. More recently, an optical sectioning tomography technique combined with light-field imaging was proposed by Zhao et al., allowing three-dimensional flame temperature distributions to be reconstructed from a single light-field camera [20]. These methods differ in their technical approaches and can be broadly categorized into two classes: one centers on tomographic imaging principles, leveraging the multi-view sampling characteristics of light-field cameras to achieve cross-sectional analysis of three-dimensional space; the other focuses on constructing high-fidelity forward imaging models. In essence, both categories represent typical inverse heat conduction problems, inferring internal temperature field distributions from surface observations [21]. Their inherent ill-posedness renders solution stability precarious, so even minor noise in the measured data may induce substantial deviations in the inversion results. Ultimately, the quality of temperature field reconstruction hinges critically on the accuracy and robustness of the inversion algorithm employed.

To address the ill-posed inverse problem of temperature field reconstruction, regularization methods have become classical approaches: Tikhonov regularization effectively suppresses noise amplification by introducing smoothness constraints [22], while the LSQR algorithm efficiently solves large-scale sparse least-squares problems in an iterative manner [23]; both have been widely adopted in radiation-based temperature field reconstruction due to their high computational efficiency and straightforward implementation [24,25,26]. However, smoothness constraints tend to blur sharp features of the temperature field. The TV-ADMM method adopts total variation regularization, preserving edges and abrupt features of the temperature field through norm-constrained spatial gradients, rendering it more suitable for reconstructing non-smooth temperature distributions compared with Tikhonov-based methods [27]. Physics-informed neural networks embed the heat conduction equation into the network architecture to enable heat source inversion [28,29,30,31], yet they typically rely on extensive training data and face deployment difficulties. Consequently, many scholars have pursued algorithmic innovations to enhance reconstruction accuracy and solution robustness. A simultaneous inversion of flame temperature and absorption coefficient was achieved by Li et al. using plenoptic imaging combined with the LMBC-NNLS algorithm, and the results demonstrated the beneficial effect of boundary constraints on the light-field thermometry inverse problem [32]. Li and Liu et al. established a multi-spectral light-field imaging model based on the reverse Monte Carlo method, realizing three-dimensional temperature distribution reconstruction and uncertainty analysis for non-uniform flames [21]. In another study, Qi et al. integrated a weighted non-negative least-squares method with light-field imaging to simultaneously reconstruct flame temperature and soot volume fraction [33].

## 2. Theory and Model

### 2.1. Basic Assumptions

To ensure that the model maintains clear physical interpretability and computational tractability, the following assumptions are adopted in this work. First, the PEDOT:PSS conductive polymer gel layer is treated as a continuous medium, and its equivalent thermal diffusivity as well as the surface convective heat transfer coefficient are taken as constants within the temperature range considered in this simulation [34]. However, in practical application, PEDOT:PSS is typically used as an aqueous dispersion, and polar secondary solvents such as dimethyl sulfoxide (DMSO) and ethylene glycol (EG) are often added to tailor the film morphology and electrical performance [4,5,35]. These solvents can markedly enhance the electrical conductivity by modifying the PEDOT chain conformation and the degree of PSS phase separation, with enhancements ranging from a factor of several to as much as two orders of magnitude [4,7,36]. In addition, the PSS component is highly hygroscopic and can absorb up to 25 wt% moisture under ambient humidity [36,37]. The absorbed water molecules increase the interchain spacing between PEDOT chains and degrade the conductive pathways, thereby reducing the electrical conductivity [37,38]. These factors—including solvent type and content, water uptake, and ambient humidity—therefore alter the effective electrical conductivity, thermal conductivity, and Joule heating distribution of PEDOT:PSS. Considering the above circumstances, this work does not directly solve the full electric potential field; instead, a spatially nonuniform Joule heat source is constructed through an equivalent electric field distribution.

It should be further clarified that the objective of the present model is to reconstruct the three-dimensional dynamic temperature field and realize overheating warning, rather than to predict the macroscopic electrical characteristics of the device. Accordingly, the model does not solve the complete electric potential field distribution between the two electrodes, nor does it compute the terminal voltage, total current, or the equivalent total resistance/conductance between the two contact points. As described above, the Joule heating source is constructed from the prescribed equivalent electric-field modulation function and the local relative conductivity distribution is directly imposed as a volumetric heat source in the heat conduction equation, rather than being obtained through a coupled solution of the electrical quantities. This treatment avoids the introduction of additional electrical boundary conditions and contact resistance models, thereby allowing the model to focus on the core objective of temperature field reconstruction.

In addition, the infrared camera is not assumed to be capable of directly penetrating the material interior; rather, it acquires equivalent infrared radiation information from the surface and near-surface regions. The internal temperature field is jointly retrieved through inverse analysis combining prior knowledge of heat conduction and multi-view light-field observations. Further, the focused light-field infrared camera satisfies the linear imaging assumption, with the point spread function approximated by a depth-dependent Gaussian model, whose width is jointly determined by the base grid scale, the diffraction limit and depth-induced defocusing. The online inversion algorithm strictly adheres to the causality requirement, meaning that the reconstruction of the current frame utilizes only the current measurement and historically reconstructed frames, without resorting to future frames or ground-truth values for correction.

In this work, the temperature range is 285–430 K, with an initial temperature of 298.15 K, which covers the entire operating window of PEDOT:PSS flexible heating patches from room temperature to the overheating warning threshold. Within this range, both the electrical and thermal conductivities of PEDOT:PSS increase monotonically with temperature [7,8,10]. Existing experimental data show that the electrical and thermal conductivities of PEDOT:PSS films increase monotonically with temperature between 173 K and 373 K. From room temperature to 375 K, solvent-treated PEDOT:PSS films can reach electrical conductivities as high as 1979 S/cm [36]. Nevertheless, the relative temperature coefficients of these properties are typically on the order of $10^{-3}$ to $10^{-2}\;K^{-1}$, i.e., a few tenths of a percent to a few percent per kelvin [7,10]. If the full temperature-dependent property model is replaced by an equivalent constant approximation $\left(\alpha =1.35\times 10^{-7}\;m^{2}/s,{\;\sigma }_{0}=1.0\right)$, the systematic error introduced over 285−430 K can be estimated as follows. Taking the typical temperature coefficient of electrical conductivity of PEDOT:PSS films as 0.2−0.5%/K, the maximum relative deviation in conductivity over a temperature variation of 145 K is approximately 30−70% [8,10]. However, because the Joule heating source term $Q_{j}\propto \sigma$, the reconstruction error is jointly suppressed by the data fidelity term and the regularization constraint in the inversion algorithm. Sensitivity analysis shows that when α is varied within ±20% of its nominal value, the root mean square error (RMSE) changes by no more than 0.035 K and the mean relative error (MRE) changes by no more than 0.007 percentage points, confirming that the proposed inversion framework is highly tolerant to deviations in thermophysical properties. Therefore, the error introduced by the constant property assumption over the temperature range considered here is acceptable and does not affect the reliability of the main conclusions. For applications requiring higher accuracy, temperature-dependent material property models may be incorporated in future work [39].

It should be noted that the above model is based on effective material properties and has not been experimentally calibrated for any specific PEDOT:PSS gel formulation (e.g., solvent type and ratio, solid content, dopants). In practice, the electrical conductivity, thermal conductivity, and Joule heating distribution of PEDOT:PSS gels are jointly influenced by water content, the proportion of polar solvents such as DMSO, ambient humidity, and temperature. In the present work, these effects are lumped into the effective thermal diffusivity α and the baseline electrical conductivity $\sigma_{0}$, rather than being modeled individually. For subsequent experimental validation, the specific material formulation, water content, and ambient humidity should be recorded, and the material properties should be recalibrated accordingly. Furthermore, the opposing effects of temperature and water content on conductivity have not been explicitly modeled in the current framework, and the resulting errors have been quantitatively assessed in the temperature sensitivity analysis described above.

### 2.2. Design and Fabrication of Actuators

The three-dimensional spatial coordinates are denoted as $\left(x,y,z\right)$, where x represents the thickness (depth) direction, y the width direction and z the height direction. The computational domain is defined as:

(1) $$\begin{array}{c} \Omega =\left[0,L_{x}\right]\times \left[0,L_{y}\right]\times \left[0,L_{z}\right] \end{array}$$

with $L_{x}=0.005\;m,L_{y}=0.030\;m$, $L_{z}=0.020\;m$. The spatial discretization employs $N_{x}=8,$ $N_{y}=12,$ $N_{z}=10$, yielding a total of $N=N_{x}N_{y}N_{z}=960$ unknown voxels. The coordinates of the $\left(i,j,k\right)$ grid point are given by

(2) $$\begin{array}{c} x_{i}=\frac{(i-1)L_{x}}{N_{x}-1},\;y_{j}=\frac{(j-1)L_{y}}{N_{y}-1},\;z_{k}=\frac{(k-1)L_{z}}{N_{z}-1} \end{array}$$

The initial temperature and the ambient temperature are both set to $T_{0}=T_{amb}=298.15\;K$. To prevent non-physical temperature values, the temperature field is constrained within the range of 285 K to 430 K during numerical progression.

**Figure 1:**
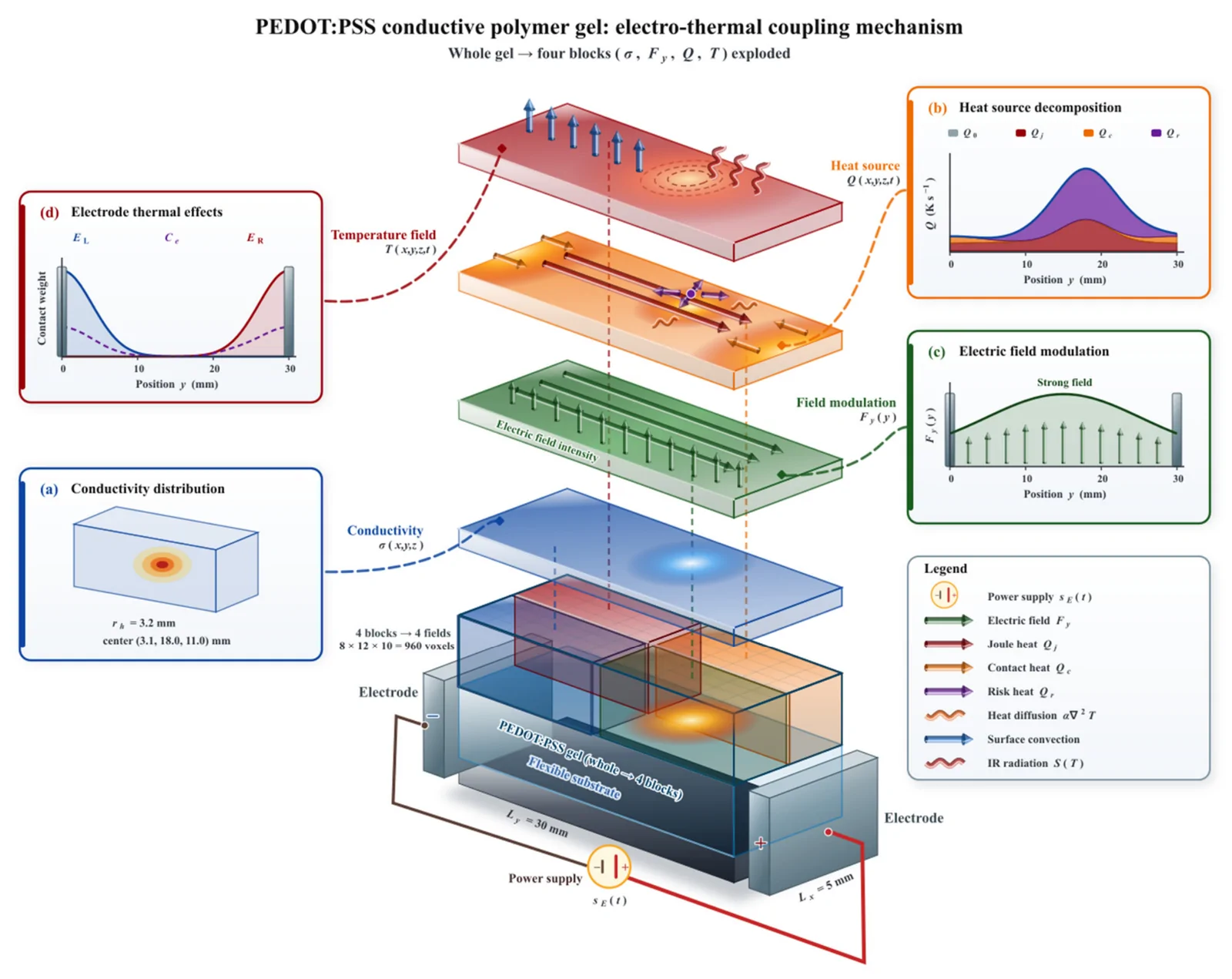
Schematic diagram of the electro-thermal coupling mechanism of the conductive polymer gel. (**a**) Nonuniform conductivity distribution induced by localized Gaussian hot spots; (**b**) heat source decomposition; (**c**) electric field modulation function along the width direction; (**d**) electrode thermal effect.

Local nonuniformity in electrical conductivity is characterized by a three-dimensional Gaussian hot spot. The primary hot spot is centered at $c_{1}=(x_{c},y_{c},z_{c})=\left(3.1,18.0,11.0\right)\;mm$, with a characteristic radius of $r_{h}=3.2\;mm$ (Figure 1a). The corresponding dimensionless hot-spot function is defined as

(3) $$\begin{array}{c} H_{1}\left(x,y,z\right)=\exp\left[-\frac{(x-x_{c}{)}^{2}+(y-y_{c}{)}^{2}+(z-z_{c}{)}^{2}}{2r_{h}^{2}}\right] \end{array}$$

The equivalent conductivity of the gel layer is then expressed as

(4) $$\begin{array}{c} \sigma \left(x,y,z\right)=\sigma_{0}\left[1+\eta_{\sigma }H_{1}\left(x,y,z\right)\right] \end{array}$$

where $\sigma_{0}=1.0$ denotes the normalized baseline electrical conductivity (dimensionless and not representing an absolute measured conductivity) and $\eta_{\sigma }=2.4$ is the conductivity enhancement coefficient. This normalization enables the Joule heating model to describe the relative spatial variation under any baseline conductivity, without being tied to the absolute conductivity measured for a specific PEDOT:PSS formulation or solvent-treatment condition. For generalization tests, a secondary hot spot $H_{2}$ can be further introduced, and a normalized composite hot-spot distribution $H=(H_{1}+w_{2}H_{2})/max(H_{1}+w_{2}H_{2})$ is constructed with a weighting factor $w_{2}$ to describe dual-hot-spot bias conditions.

The equivalent electric field is primarily distributed along the y-direction. Considering that the electric field is stronger in the central electrode region and weaker near the edges, the dimensionless electric field modulation function (Figure 1c) is defined as

(5) $$\begin{array}{c} F_{y}\left(y\right)=0.72+0.28exp\left[-\frac{(y-L_{y}/2{)}^{2}}{2(0.34L_{y}{)}^{2}}\right] \end{array}$$

The contact thermal effects at the two terminal electrodes are described by the left and right electrode attenuation functions, respectively:

(6) $$\begin{array}{c} E_{L}\left(y\right)=\exp\left(-\frac{y^{2}}{2w_{e}^{2}}\right),\;E_{R}\left(y\right)=\exp\left(-\frac{(y-L_{y}{)}^{2}}{2w_{e}^{2}}\right) \end{array}$$

where $w_{e}=0.004\;m$ is the electrode influence bandwidth (Figure 1d). Combining hot-spot enhancement and electrode boundary effects, the spatial weighting function for the contact heat source is defined as:

(7) $$\begin{array}{c} C_{e}\left(x,y,z\right)=\frac{1}{2}\left[E_{L}\left(y\right)+E_{R}\left(y\right)\right]\left[0.35+0.65H_{1}\left(x,y,z\right)\right] \end{array}$$

In this work, the heat source terms are normalized by the volumetric heat capacity to represent the equivalent temperature rise rate. The Joule heat source $Q_{j}$ and the electrode contact heat source $Q_{c}$ are respectively expressed as

(8) $$\begin{array}{c} Q_{j}\left(x,y,z,t\right)=k_{j}s_{E}^{2}\left(t\right)\frac{\sigma \left(x,y,z\right)}{\sigma_{0}}F_{y}\left(y\right) \end{array}$$

(9) $$\begin{array}{c} Q_{c}\left(x,y,z,t\right)=k_{c}s_{H}\left(t\right)C_{e}\left(x,y,z\right) \end{array}$$

where $k_{j}=1.15$, $k_{c}=5.8$ are the intensity coefficients for the Joule and contact heat sources, respectively. $s_{E}(t)$ denotes the temporal modulation function of the equivalent electric field strength, and $s_{H}(t)$ denotes the temporal modulation function of the electrode contact heat source.

The heating process is divided into five stages: initial uniform stage, electrode energization heating stage, electrothermal hot-spot formation stage, thermal diffusion stage and overheating risk stage. The corresponding $s_{E}(t)$, $s_{H}(t)$ and the overheating expansion factor $s_{R}(t)$ are specified as follows:

For 0≤t<2 s: $s_{E}(t)=0.20+0.20t/2$, $s_{H}(t)=0$, $s_{R}(t)=1.0$;

For 2≤t<4 s: $s_{E}(t)=0.58+0.18(t-2)/2$, $s_{H}(t)=0.18$, $s_{R}(t)=1.0$;

For 4≤t<7 s: $s_{E}(t)=0.82$, $s_{H}(t)=0.42+0.30(t-4)/3$, $s_{R}(t)=1.0+0.10(t-4)$;

For 7≤t<8.5 s: $s_{E}(t)=0.92$, $s_{H}(t)=0.72$, $s_{R}(t)=1.25+0.16(t-7)$;

For 8.5≤t≤10 s: $s_{E}(t)=1.0$, $s_{H}(t)=0.82$, $s_{R}(t)=1.45+0.18(t-8.5)$.

During the overheating risk stage, an intensifying and spatially expanding risk heat source is introduced:

(10) $$\begin{array}{c} Q_{r}(x,y,z,t)=k_{r}\max(0,t-8.5{)}^{1.15}\exp\left[-\frac{r^{2}}{2(r_{h}s_{R}(t){)}^{2}}\right] \end{array}$$

where $k_{r}=14.0$, $r^{2}=(x-x_{c}{)}^{2}+(y-y_{c}{)}^{2}+(z-z_{c}{)}^{2}$. The total heat source is thus given by:

(11) $$\begin{array}{c} Q\left(x,y,z,t\right)=Q_{0}+Q_{j}\left(x,y,z,t\right)+Q_{c}\left(x,y,z,t\right)+Q_{r}\left(x,y,z,t\right) \end{array}$$

where $Q_{0}=0.18$ denotes the background heat source intensity (Figure 1b).

The temperature field evolution satisfies the three-dimensional heat conduction equation with volumetric heat generation and surface convective heat dissipation:

(12) $$\begin{array}{c} \frac{\partial T}{\partial t}=\alpha \nabla^{2}T+Q-h_{c}M_{s}\left(T-T_{amb}\right) \end{array}$$

where $T\left(x,y,z,t\right)$ is the temperature field, $M_{s}$ is the surface voxel mask, $\alpha_{0}=1.35\times 10^{-7}\;m^{2}\;s^{-1}$ is the baseline effective thermal diffusivity adopted in the present numerical model, and $h_{c}=0.018\;s^{-1}$ is the equivalent surface cooling rate normalized by the volumetric heat capacity. The latter should not be confused with the conventional convective heat transfer coefficient, which carries units of $W\;m^{-2}\;K^{-1}$. It is important to emphasize that $\alpha_{0}$ and $h_{c}$ are treated as constant parameters within the current equivalent numerical framework as a simplification. In real PEDOT:PSS gel systems, these parameters may vary with material formulation, moisture content, and temperature. The baseline value $\alpha_{0}=1.35\times 10^{-7}\;m^{2}\;s^{-1}$ is used solely for numerical demonstration and is not claimed to be a measured material constant for any specific PEDOT:PSS formulation. Notably, this value falls within the typical range of effective thermal diffusivities reported for polymer-based composites, which can be reliably characterized using cost-effective measurement techniques such as the one described by Janek et al. [40], where an accuracy of approximately 5% was achieved for polyurethane composites. To quantify the sensitivity of the reconstruction results to uncertainty in the thermal diffusivity, a parameter mismatch experiment is conducted. The inversion model is fixed at $\alpha =\alpha_{0}$, while the true α used in the forward model is varied over $\left\{0.8\alpha_{0},0.9\alpha_{0},\;1.0\alpha_{0},\;1.1\alpha_{0},\;1.2\alpha_{0}\right\}$, with all other inversion parameters (λ, μ, etc.) held frozen. Over the examined ±20% range of α mismatch, the reconstruction accuracy remains largely stable: the maximum variations in RMSE and MRE are 0.9575% and 0.7193%, respectively; the Dice coefficient varies by at most 0.0144; the hotspot localization error changes by only 0.000741 mm; and the warning time deviation remains at −1 s across all cases. These results confirm that the proposed inversion framework is weakly sensitive to α uncertainties within the considered range.

**Figure 2:**
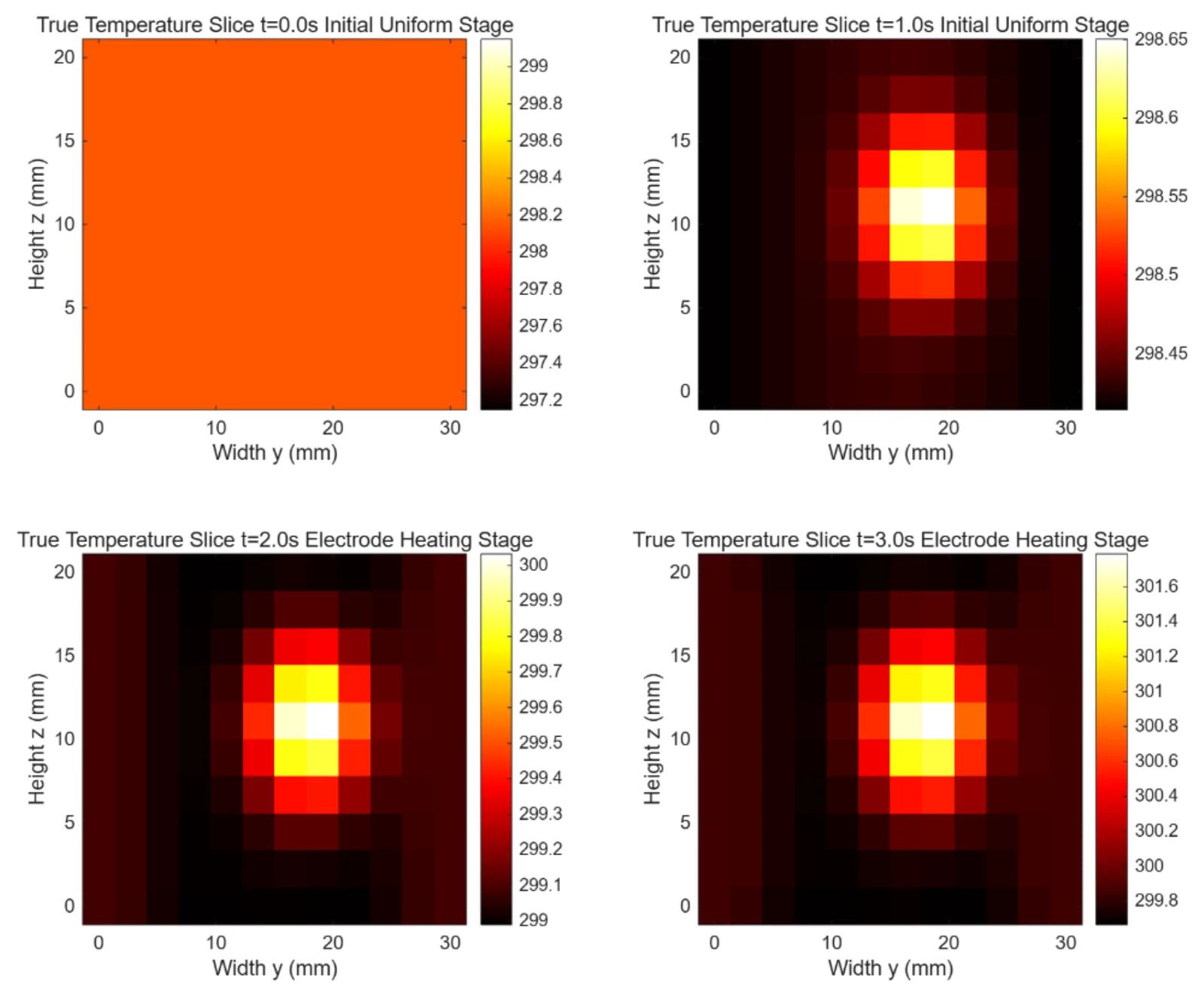
Ground-truth distributions of the cross-sectional temperature field at different temporal instants.

Zero normal heat flux boundary conditions are imposed as

(13) $$\begin{array}{c} \nabla T\cdot n=0,\;\left(x,y,z\right)\in \partial \Omega \end{array}$$

where n denotes the outward normal vector on the boundary. The time step is Δt=1 s, with a total simulation duration of 10 s, comprising 11 temporal instants. Slices of the ground-truth temperature field at four representative instants are shown in Figure 2, corresponding to t=0.0 s (initial uniform stage), t=2.0 s (electrode energization heating stage), t=4.0 s (electrothermal hot-spot formation stage) and t=9.0 s (overheating risk stage).

### 2.3. Forward Model of Focused Light-Field Infrared Imaging

**Figure 3:**
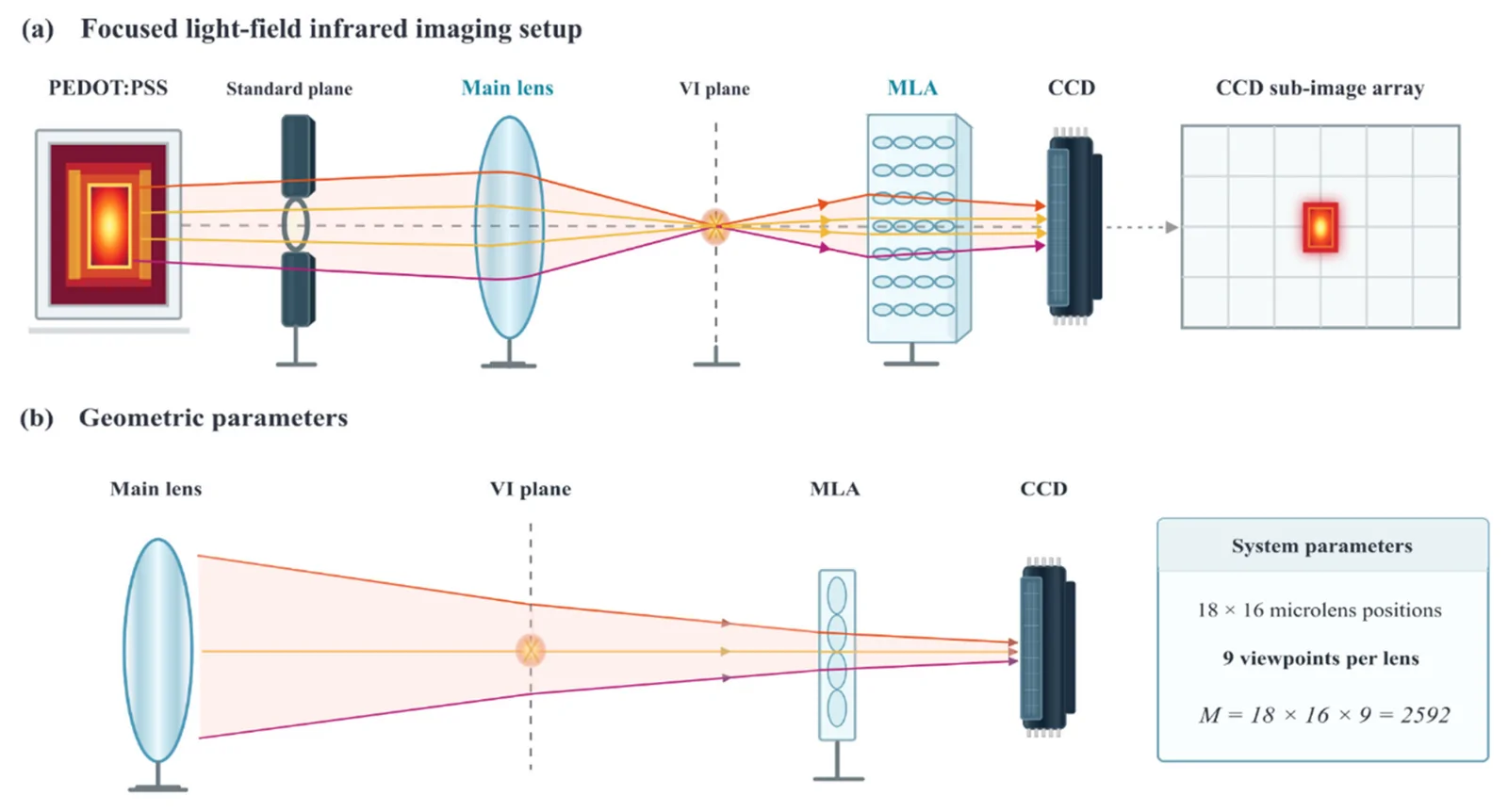
Schematic diagram of the focused light-field infrared imaging mechanism. (**a**) Schematic of the light-field infrared imaging simulation setup; (**b**) optical path and measurement parameters of the imaging system.

Unlike conventional infrared imaging techniques, the focused light-field infrared camera in this paper enables dense acquisition of radiance not only in the spatial domain but also in the angular domain. The focused light-field infrared camera is composed of a main lens, an internal microlens array (MLA) and a CCD sensor. A virtual image (VI) plane exists between the main lens plane and the MLA plane. The infrared rays emitted from the conductive polymer gel heating patch are converged by the main lens onto the virtual image plane, then projected onto the corresponding microlens, where they are converged again and cast onto the CCD sensor (Figure 3a). The key geometric parameters of the imaging system employed in this work is listed (Table 1). The infrared imaging model transforms the three-dimensional temperature field into multi-channel infrared light-field observations. According to the Stefan–Boltzmann law, the equivalent infrared radiation source term corresponding to the temperature field is given by:

(14) $$\begin{array}{c} S\left(T\right)=\varepsilon \sigma_{SB}T^{4} \end{array}$$

where S denotes the equivalent radiation source term, ε=0.92 is the equivalent surface emissivity of the material and $\sigma_{SB}=5.670374419\times 10^{-8}\;W\cdot \;m^{-2}\cdot \;K^{-4}$ is the Stefan-Boltzmann constant. Once S is retrieved through inversion, the temperature field can be recovered from the following relation:

(15) $$\begin{array}{c} T={\left(\frac{S}{\varepsilon \sigma_{SB}}\right)}^{1/4} \end{array}$$

A focused light-field infrared camera forms its observation channels through the combined spatial sampling of the microlens array and angular sampling. In this work, an 18 × 16 microlens array is adopted, with each microlens containing 9 angular views. The total number of measurement channels is thus M=18×16×9=2592 (Figure 3b). Given that the number of unknown voxels is N=960, the system matrix satisfies $A\in R^{2592\times 960}$. Consequently, this problem constitutes an overdetermined, sparse and ill-posed inverse problem, and the number of measurements exceeds the number of unknowns. For the m-th measurement channel and the j-th voxel, the element of the system matrix is defined as:

(16) $$\begin{array}{c} A_{mj}=G\exp\left(-\mu_{eff}x_{j}\right)\left[1-\exp\left(-\mu_{eff}\Delta x\right)\right]w_{ang,m}w_{psf,mj} \end{array}$$

where G=1 is the combined coefficient of sensor gain and exposure time, $\mu_{eff}=60\;m^{-1}$ is the equivalent near-surface attenuation coefficient, Δx is the grid spacing in the thickness direction, $w_{ang,m}$ denotes the angular view weight and $w_{psf,mj}$ represents the depth-dependent point spread function weight. It should be noted that the depth-dependent term $\exp\left(-\mu_{eff}\Delta x\right)$ in Equation (16) does not imply that the infrared radiation penetrates into the gel bulk and is detected from internal depths. Instead, it denotes the equivalent measurement sensitivity of the focused light-field imaging system to thermal states at varying near-surface depths, arising from the depth-varying point spread function and the optical system’s response to shallow subsurface thermal distributions. In this sense, Equation (16) constitutes an equivalent forward imaging model rather than a full solution to the radiative transfer equation.

The point spread function is approximated by a Gaussian model, whose lateral width increases with the degree of depth defocus:

(17) $$\begin{array}{c} \sigma_{y}\left(x\right)=\sigma_{z}\left(x\right)=\sigma_{0}\left[1+k_{d}\frac{\left|x-x_{f}\right|}{\frac{L_{x}}{2}+\epsilon_{0}}\right] \end{array}$$

where $\sigma_{0}$ is the baseline PSF width, $k_{d}$ is the defocus broadening coefficient, $x_{f}$ is the focal depth and $\epsilon_{0}$ is a small constant introduced to prevent division by zero. The diffraction scale of the main lens is governed by the infrared central wavelength $\lambda_{IR}=10\mu m$, and influences the baseline spot width through the f-number $F^{\#}$.

Under noise-free conditions, the light-field measurement vector $b_{clean}\in R^{M}$ and the discretized radiation source vector $s\in R^{N}$ satisfy the linear relationship:

(18) $$\begin{array}{c} b_{clean}=As. \end{array}$$

To compensate for gain discrepancies among different measurement channels, the system matrix A is subjected to row-wise normalization in this work. With infrared measurement noise taken into account, the observation vector is expressed as

(19) $$\begin{array}{c} b=\max\left[0,\;b_{clean}+\eta max(b_{clean})\xi \right], \end{array}$$

where η denotes the relative noise level and ξ represents a random noise vector following the standard normal distribution. In this work, the noise level is varied as $\eta \in \left\{0,0.01,0.03,0.05,0.07\right\}$, with η=0.03 adopted as the baseline case for the primary operating condition.

### 2.4. ETP-Causal LSQR

The unknown variable in the inversion model is not the temperature T itself, but rather the equivalent infrared radiation source term s. Once s is retrieved, the three-dimensional temperature field is subsequently recovered by Equation (15). The conventional Tikhonov-LSQR inversion can be formulated as

(20) $$\begin{array}{c} \underset{s}{min}{∥As-b∥}_{2}^{2}+\lambda {∥Ls∥}_{2}^{2} \end{array}$$

where λ denotes the Tikhonov regularization parameter and L is the spatial smoothing operator. Tikhonov-LSQR is a classical regularization baseline method that introduces a spatial smoothness regularization term in addition to the data fidelity term, suppressing noise amplification in the ill-posed inverse problem through an L2-norm constraint. While this approach benefits from high computational efficiency and straightforward implementation, its frame-by-frame independent processing with only spatial smoothing constraints tends to blur sharp features of the temperature field and fails to exploit temporal evolution information. Consequently, it often yields unstable reconstructions, particularly in deeper regions.

To enhance the physical consistency of online inversion, this work proposes the ETP-Causal LSQR model. The core idea is to leverage the reconstructed temperature field from the previous time step to predict the prior temperature of the current time step via the heat conduction equation, and to incorporate this prior into the LSQR inversion as a soft constraint. $T_{rec}^{n-1}$ denotes the reconstructed temperature field at the (n − 1) frame. The thermal prediction for the current frame is then given by:

(21) $$\begin{array}{c} T_{pred}^{n}=clip\left[T_{rec}^{n-1}+\Delta t\;\alpha \nabla^{2}T_{rec}^{n-1}\right] \end{array}$$

where $clip\left(\cdot \right)$ denotes the temperature range truncation operation, which confines the predicted temperature field within physically admissible bounds. The corresponding radiation source prior is then obtained as:

(22) $$\begin{array}{c} s_{pred}^{n}=\varepsilon \sigma_{SB}{\left(T_{pred}^{n}\right)}^{4} \end{array}$$

Finally, the optimization objective of the ETP-Causal LSQR model is defined as:

(23) $$\begin{array}{c} \underset{s^{n}}{min}{∥As^{n}-b^{n}∥}_{2}^{2}+\lambda {∥Ls^{n}∥}_{2}^{2}+\mu {∥s^{n}-s_{pred}^{n}∥}_{2}^{2} \end{array}$$

where $b^{n}$ is the infrared light-field observation at the current time instant, λ is the spatial regularization weight and μ is the electro-thermal physical prior weight.

Equation (23) can be equivalently reformulated as an augmented least-squares system:

(24) $$\left[\begin{array}{c} A\\ \sqrt{\lambda }L\\ \sqrt{\mu }I \end{array}\right]s^{n}=\left[\begin{array}{c} b^{n}\\ 0\\ \sqrt{\mu }s_{pred}^{n} \end{array}\right]$$

where I denotes the identity matrix. In this work, the parameters are fixed as λ=3.16228, μ=0.001, which are determined from the calibration case. It should be emphasized that the ETP-Causal LSQR method relies solely on the current measurement $b^{n}$ and the reconstructed temperature field from the previous time step $T_{rec}^{n-1}$ without resorting to future frames or ground-truth values. Therefore, it strictly satisfies the causality requirement for online overheating warning.

For comparison, this work also considers offline thermal prior LSQR and TV-ADMM methods. The former incorporates non-causal priors constructed from both preceding and subsequent frames, serving merely as an upper performance bound for offline reconstruction. The latter suppresses artifacts through total variation regularization, whose optimization formulation is given by

(25) $$\begin{array}{c} \underset{s}{min}{∥As-b∥}_{2}^{2}+\beta {∥Gs∥}_{1} \end{array}$$

where β is the TV regularization parameter and G denotes the spatial gradient operator. TV-ADMM replaces the L2 smoothness prior with total variation regularization. By imposing sparsity on the gradient field through an L1-norm penalty, this method suppresses artifacts while preserving sharp edges and discontinuities in the temperature field, making it more suitable than Tikhonov regularization for reconstructing non-smooth temperature distributions. However, TV-ADMM remains a frame-by-frame spatial-domain regularization method, and solving the L1-norm optimization problem via the alternating direction method of multipliers typically requires numerous iterations, resulting in a relatively high computational cost. It is adopted here as a comparison method to assess the performance difference between edge-preserving regularization and electro-thermal physical prior constraints.

### 2.5. Parameter Calibration, Evaluation Metrics and Overheating Warning

To prevent information leakage from the test set, a calibration/test splitting strategy is adopted in this work. Regularization parameters are searched exclusively on the calibration case. The fixed parameter sets are then applied to the primary operating condition, noise-level scanning cases, ideal (noise-free) case and generalization cases without being retuned based on the test ground truth. The grid search range for the regularization parameters is set as follows: the spatial smoothing weight λ is uniformly sampled on a logarithmic scale over the range $10^{-4}$ to $10^{2}$ with 9 discrete values and the heat conduction constraint weight μ is uniformly sampled on a logarithmic scale over the range $10^{-3}$ to $10^{2}$ with 8 discrete values, yielding a total of 72 candidate parameter combinations. The comprehensive scoring function is defined as

(26) $$\begin{array}{c} Score=MRE+0.030\;RMSE+4.000\left(1-Dice\right) \end{array}$$

where the root mean square error (RMSE), mean absolute error (MAE) and mean relative error (MRE) are respectively employed to evaluate the global reconstruction error:

(27) $$\begin{array}{c} RMSE=\sqrt{\frac{1}{N}\sum_{j=1}^{N}{\left(T_{rec,j}-T_{true,j}\right)}^{2}} \end{array}$$

(28) $$\begin{array}{c} MAE=\frac{1}{N}\sum_{j=1}^{N}\left|T_{rec,j}-T_{true,j}\right| \end{array}$$

(29) $$\begin{array}{c} MRE=\frac{1}{N}\sum_{j=1}^{N}\frac{\left|T_{rec,j}-T_{true,j}\right|}{T_{true,j}} \end{array}$$

RMSE, MAE and MRE characterize the reconstruction accuracy from different perspectives: MAE directly quantifies the average absolute deviation between the reconstructed and true temperatures per voxel, reflecting the overall mean deviation of the reconstruction on a global scale; RMSE, by assigning higher weights to larger deviations, is particularly sensitive to localized severe discrepancy regions within the reconstruction; and MRE normalizes the absolute error as a percentage relative to the true temperature, thereby eliminating the influence of magnitude disparities across different temperature ranges and providing a fair assessment of reconstruction accuracy in low-temperature regions.

To evaluate the localization capability of the high-temperature core region, this work adopts a relative temperature-rise threshold to define the high-temperature zone. $T_{max}$ denotes the maximum temperature of the true temperature field at the current time instant. The high-temperature threshold is then defined as

(30) $$\begin{array}{c} T_{hot}=T_{amb}+0.8\left(T_{max}-T_{amb}\right) \end{array}$$

The high-temperature region mask is defined as $M_{hot}=\{T\geq T_{hot}\}$. Based on this mask, the Dice coefficient and intersection over union (IoU) coefficient are respectively defined as

(31) $$\begin{array}{c} Dice=\frac{2|M_{true}\cap M_{rec}|}{\left|M_{true}|+|M_{rec}\right|} \end{array}$$

(32) $$\begin{array}{c} IoU=\frac{\left|M_{true}\cup \;M_{rec}\right|}{\left|M_{true}\cap \;M_{rec}\right|} \end{array}$$

To further evaluate whether the reconstructed results conform to the thermal diffusion law, the instantaneous heat conduction prediction residual is defined as

(33) $$\begin{array}{c} R_{\mathrm{heat}}^{n}\left(x_{i,j}\right)=T_{\mathrm{rec}}^{n}\left(x_{i,j}\right)-\left[T_{\mathrm{rec}}^{n-1}\left(x_{i,j}\right)+\Delta t\cdot \alpha \nabla^{2}T_{\mathrm{rec}}^{n-1}\left(x_{i,j}\right)\right] \end{array}$$

Based on the instantaneous heat conduction prediction residual defined in Equation (33), the following physical consistency evaluation metrics are further constructed: the mean absolute thermal residual $R_{\mathrm{MA}}$, the root mean square thermal residual $R_{\mathrm{RMS}}$ and the normalized thermal residual $R_{N}$ is defined as

(34) $$\begin{array}{c} R_{MA}=\frac{1}{N_{x}N_{y}N_{t}}\;\sum_{i=1}^{N_{x}}\sum_{j=1}^{N_{y}}\sum_{n=1}^{N_{t}}\left|R_{\mathrm{heat}}^{n}\left(x_{i,j}\right)\right|\; \end{array}$$

(35) $$\begin{array}{c} R_{RMS}=\sqrt{\frac{1}{N_{x}N_{y}N_{t}}\;\sum_{i=1}^{N_{x}}\sum_{j=1}^{N_{y}}\sum_{n=1}^{N_{t}}{\left[R_{\mathrm{heat}}^{n}\left(x_{i,j}\right)\right]}^{2}\;} \end{array}$$

(36) $$\begin{array}{c} R_{N}=\frac{R_{RMS}}{\frac{\Delta T_{max}}{\Delta t}} \end{array}$$

The overheating warning is collectively determined by three criteria: the maximum temperature, the maximum heating rate and the high-temperature volume fraction:

(37) $$\begin{array}{c} T_{max}\left(t\right)=\underset{\Omega }{max}T\left(x,y,z,t\right) \end{array}$$

(38) $$\begin{array}{c} R_{T}\left(t\right)=\frac{T_{max}\left(t\right)-T_{max}\left(t-\Delta t\right)}{\Delta t} \end{array}$$

(39) $$\begin{array}{c} V_{hot}\left(t\right)=\frac{1}{N}\sum_{j=1}^{N}\Gamma \left[T_{j}\left(t\right)\geq T_{volume}\right] \end{array}$$

where $R_{T}(t)$ denotes the maximum temperature rise rate at time t, $V_{hot}(t)$ the high-temperature volume fraction and $\Gamma \left[\cdot \right]$ the indicator function. $\Gamma \left[\cdot \right]$ denotes the indicator function, which equals 1 if the condition inside the brackets is satisfied and 0 otherwise. The final warning time is then defined as

(40) $$\begin{array}{c} t_{warn}=\min t \end{array}$$

(41) $$\begin{array}{c} s.t.\;\;\left\{T_{max}(t)\geq T_{overheat}\;or\;R_{T}(t)\geq R_{th}\;or\;V_{hot}(t)\geq V_{th}\right\} \end{array}$$

The warning thresholds adopted in this work are $T_{\mathrm{overheat}}=345\;K$ for the maximum temperature, $T_{\mathrm{volume}}=338\;K$ for the high-temperature volume fraction threshold, $V_{\mathrm{th}}=0.018$ for the volume fraction threshold and $R_{\mathrm{th}}=4.2\;K\cdot s^{-1}$ for the maximum temperature rise rate threshold. It should be noted that the aforementioned $T_{\mathrm{overheat}}=345\;K$ and $T_{\mathrm{volume}}=338\;K$ are synthetic trigger temperatures artificially defined within the numerical simulation framework of this work. They do not represent the critical temperature for human skin safety or the threshold for tissue thermal damage. To quantify the accumulated thermal exposure associated with the present high-temperature scenarios, we perform an additional analysis based on the CEM43 (Cumulative Equivalent Minutes at 43 °C) thermal dose model [41]. Integrating the complete 0–10 s temperature history, the maximum cumulative CEM43 dose reaches $3.9293\times 10^{8}$ min for the true temperature field and $8.5421\times 10^{7}$ min for the ETP-Causal reconstructed field. These values are orders of magnitude higher than the widely recognized thresholds for irreversible tissue damage (typically CEM43 > 120 min or 240 min), confirming that the present simulation conditions correspond to a severe overheat scenario rather than a realistic skin contact safety boundary. This further justifies that the adopted thresholds are used solely for algorithm validation purposes and should not be misconstrued as safety limits for practical wearable applications. A comprehensive evaluation of skin-contact thermal safety would require the integration of time-temperature damage models, which is beyond the scope of the present work. However, it should be noted that this multi-criteria strategy enables simultaneous response to three types of risk signals: absolute overheating, rapid temperature rise and local high-temperature volume expansion.

**Figure 4:**
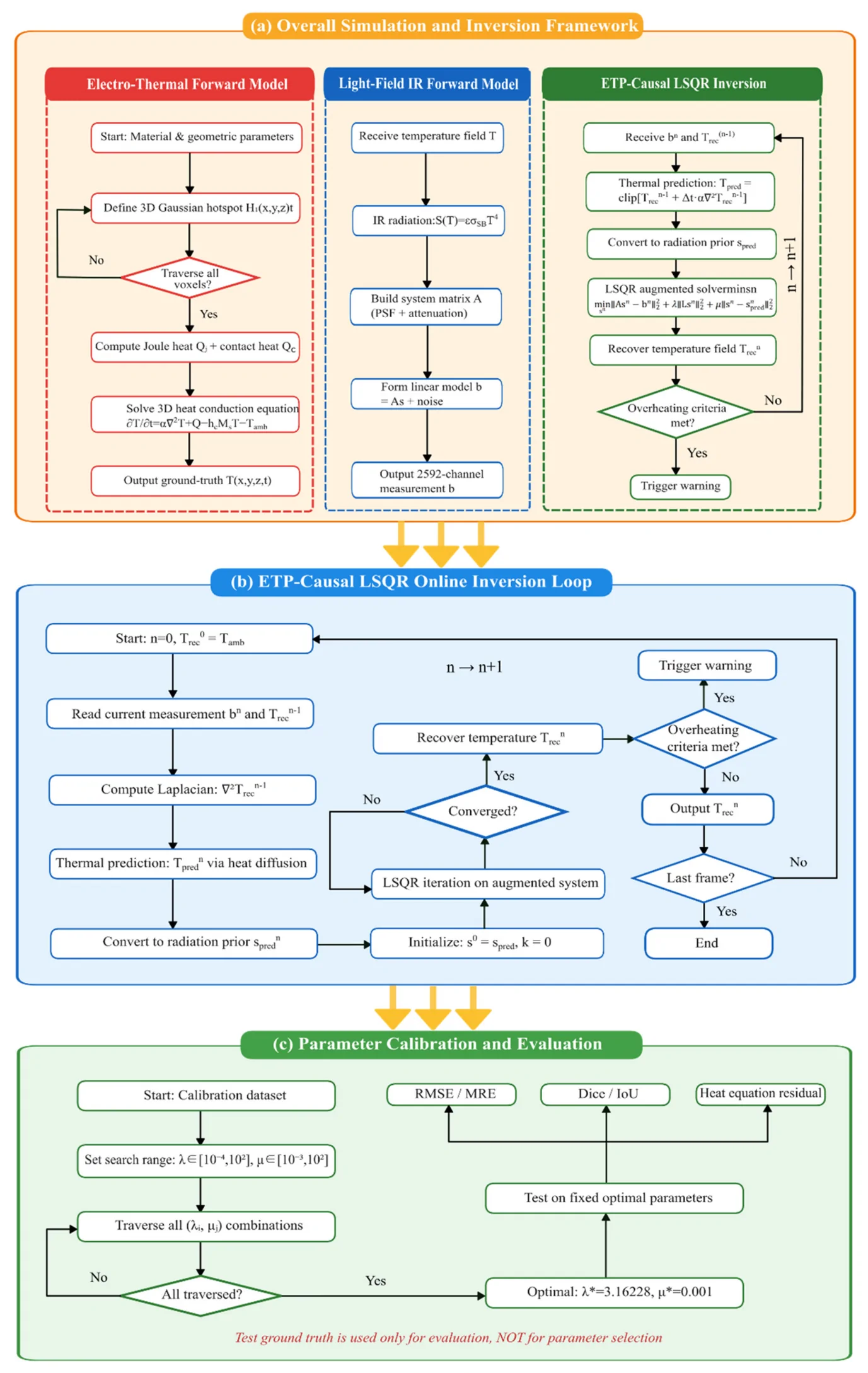
Overall flowchart of the proposed algorithm. (**a**) Overall simulation and inversion framework; (**b**) iterative logic of the ETP Causal LSQR inversion; (**c**) calibration strategy for regularization parameters.

To clearly illustrate the complete logical chain of the aforementioned forward electro-thermal coupled temperature field model, forward focused light-field infrared imaging model and the ETP-Causal LSQR inversion-based warning method, Figure 4 presents the overall system flowchart of the proposed algorithm. The overall simulation and inversion framework is shown in Figure 4a. The detailed illustration of the iterative logic of ETP-Causal LSQR in single-frame inversion is provided in Figure 4b and the calibration strategy for the regularization parameters λ and μ is presented in Figure 4c, along with the multi-dimensional evaluation workflow.

## 3. Results and Discussion

### 3.1. Parameter Calibration and Performance Under the Ideal Scenario

**Figure 5:**
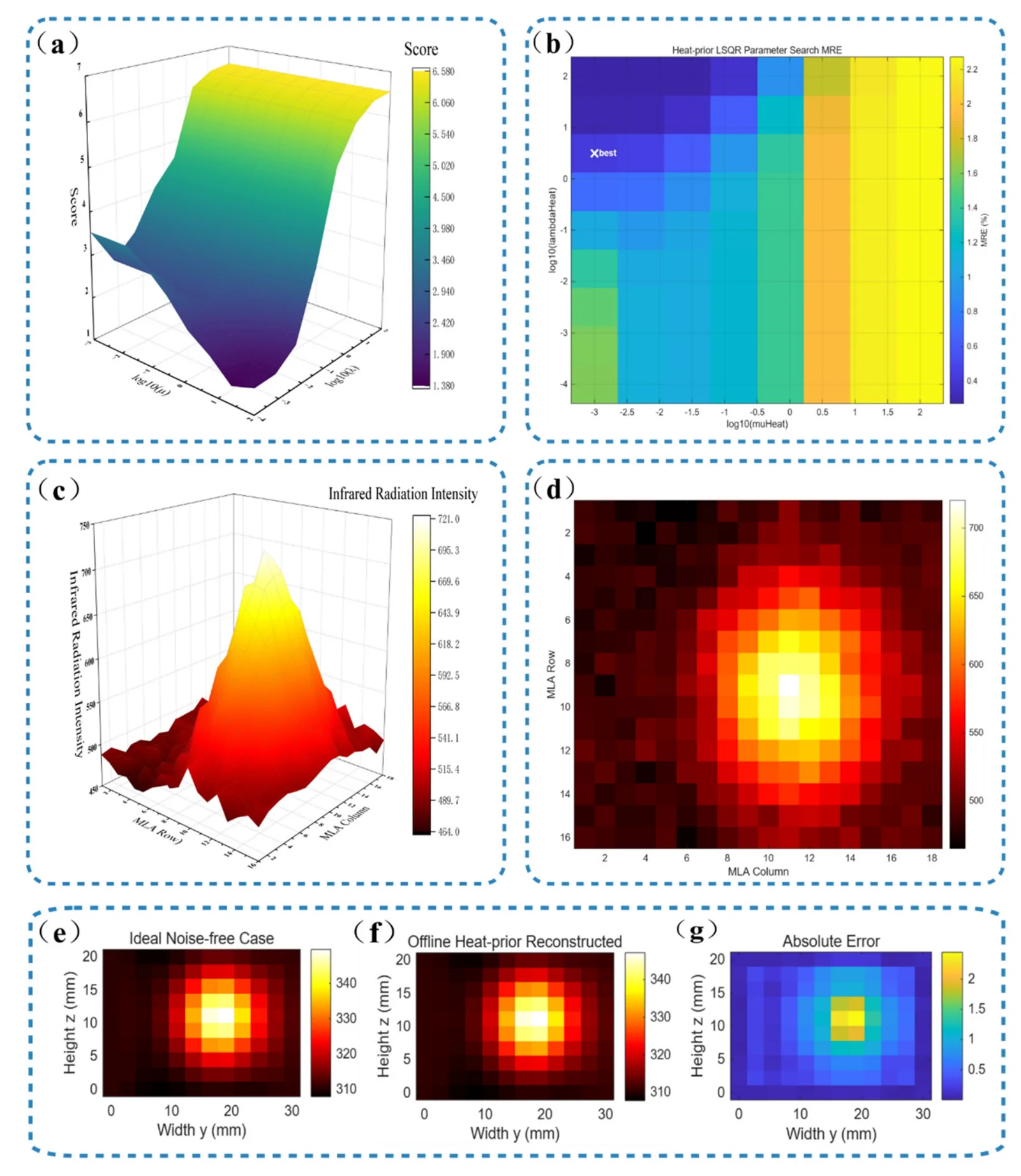
Schematic diagrams of parameter calibration and reconstruction under the ideal (noise-free) scenario. (**a**) Comprehensive scores of parameter combinations; (**b**) mean relative errors of parameter combinations; (**c**) three-dimensional radiation intensity distribution; (**d**) two-dimensional infrared light-field measurement image; (**e**) ground-truth temperature field of the target cross-section; (**f**) temperature field reconstructed by the offline heat prior LSQR method; (**g**) reconstruction error of the heat prior algorithm.

The selection of regularization parameters critically determines the accuracy and stability of the inverse solution. In this work, a grid search strategy is adopted to calibrate the spatial smoothing weight λ and the heat conduction constraint weight μ. The search ranges are $\lambda \in \left[10^{-4},10^{2}\right]$ (9 points on a logarithmic scale) and $\mu \in \left[10^{-3},10^{2}\right]$ (8 points on a logarithmic scale), yielding a total of 72 candidate parameter combinations. The calibration is performed using the comprehensive scoring function $\mathrm{Score}=\mathrm{MRE}+0.030\;\mathrm{RMSE}+4.000\;\left(1-\mathrm{Dice}\right)$, where a lower score indicates a more accurate parameter selection. Figure 5a presents the three-dimensional scoring distribution heatmap for the 72 parameter combinations on the calibration case and Figure 5b shows the corresponding mean relative errors (MRE) for each combination. It is observed that the MRE distribution in the parameter space exhibits distinct zonal characteristics. When the heat conduction constraint weight μ is excessively large, the physical prior constraint becomes overly dominant, suppressing local detail features of the true temperature field and resulting in oversmoothed reconstructions with MRE exceeding 2%. Conversely, when μ is set to a sufficiently small value, the overall MRE remains at a relatively low level and the reconstruction performance remains stable. The influence of the spatial smoothing weight λ on reconstruction accuracy is comparatively milder. However, an excessively small λ can lead to insufficient regularization and exacerbates the ill-posedness of the inverse problem, whereas an overly large λ induces oversmoothing. The optimal parameter point is located at λ=3.162 and μ=0.001, yielding a comprehensive score of 1.397, which corresponds to the global minimum (Table 2). All subsequent experiments in this section are conducted with this fixed set of calibrated parameters to ensure fair comparison.

Figure 5c,d, respectively, present the simulated three-dimensional radiation intensity distribution and the two-dimensional infrared light-field measurement image generated by the forward model of the focused light-field infrared camera at t=10 s under the primary operating condition. The grayscale values at the 18×16 microlens positions in Figure 5d reflect the equivalent infrared radiation intensity at the material surface and near-surface regions from different viewing angles. It is evident that the microlens positions corresponding to the hot-spot center exhibit pronounced radiation enhancement, while the surrounding regions show relatively lower radiation intensity, thereby validating the effective imaging capability of the forward model for nonuniform temperature fields. This measurement image will serve as the input to the subsequent inversion algorithm.

To verify the theoretical performance upper bound of the thermal prior model, reconstruction tests are first conducted under the ideal noise-free condition. Under this scenario, the accuracy metrics of the ETP-Causal LSQR and the offline heat prior LSQR are numerically indistinguishable to three decimal places (Table 3). It should be noted that this agreement does not imply that the two methods produce strictly identical temperature fields. A per-frame diagnostic comparison shows that the two priors differ by up to 12.35 K, but after LSQR inversion, the maximum absolute difference between the final reconstructed fields is only 0.178 K, with an RMSE of 0.020 K. The global metrics—RMSE, MAE, MRE, Dice, and IoU—are insensitive to such small differences (<0.18 K per voxel), and the three-decimal display precision further masks the discrepancy. The causal online method therefore achieves effectively the same reconstruction accuracy as the non-causal offline upper bound under the reported precision, while strictly respecting causality.

Figure 5e–g present the ground-truth temperature field slice of the target cross-section, the reconstruction result obtained by the offline heat prior LSQR algorithm and the corresponding absolute error distribution under the ideal noise-free condition, respectively. Overall, the reconstructed temperature field exhibits strong spatial consistency with the ground truth, as shown in Figure 5e,f The error distribution reveals that the maximum error is concentrated in the hot-spot core region and decreases rapidly toward the surrounding low-temperature areas (Figure 5g), with an overall MRE of only 0.161%.

Notably, even under ideal noise-free conditions, the reconstruction error is not identically zero everywhere; instead, it remains concentrated in the core region of the hotspot. This phenomenon does not arise from measurement noise but rather from the inherent ill-posedness of the inverse problem: the inverse heat conduction problem is mathematically a typical inverse source problem for elliptic equations, in which high-frequency components are naturally attenuated in the forward mapping and cannot be fully recovered even with regularization constraints during inversion. Although the MRE is only 0.161%, the localized errors in the hotspot region indicate a physical trade-off between the sharpness of the heat source distribution and the spatial resolution of the inversion—an overly strong prior constraint tends to smooth the hotspot, whereas an overly weak one introduces boundary oscillations [42]. This trade-off is further amplified in the subsequent noisy cases.

### 3.2. Reconstruction Performance and Ablation Study Under the Primary Operating Condition

**Figure 6:**
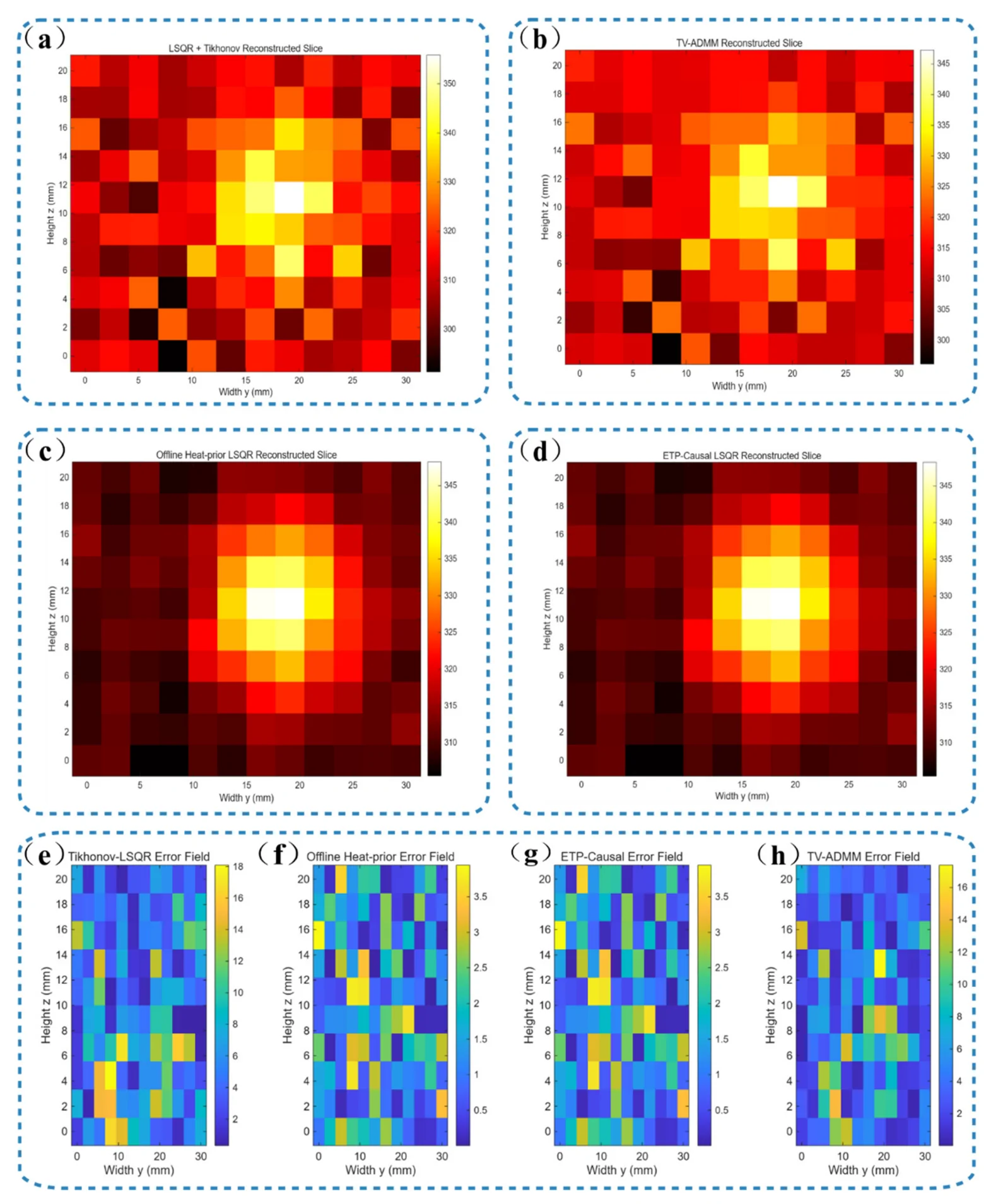
Comparison of reconstructed temperature fields and error distributions for various inversion algorithms. (**a**) reconstructed temperature field by Tikhonov LSQR; (**b**) reconstructed temperature field by TV ADMM; (**c**) reconstructed temperature field by offline heat prior LSQR; (**d**) reconstructed temperature field by ETP Causal LSQR; (**e**) absolute error distribution of Tikhonov LSQR; (**f**) absolute error distribution of offline heat prior LSQR; (**g**) absolute error distribution of ETP Causal LSQR; (**h**) absolute error distribution of TV ADMM.

To validate the performances of each module in the inversion algorithm, ablation experiments are conducted under the primary operating condition (η=0.03). The compared algorithms include: Tikhonov-LSQR (baseline), spatial heat prior only (ablation), offline heat prior LSQR (offline upper bound), ETP-Causal LSQR (proposed) and TV-ADMM (comparison). Table 4 lists the comprehensive performance comparison of all algorithms at t=10 s under the primary operating condition. Figure 6 presents the reconstructed temperature fields and corresponding error distributions for each inversion algorithm.

**Figure 7:**
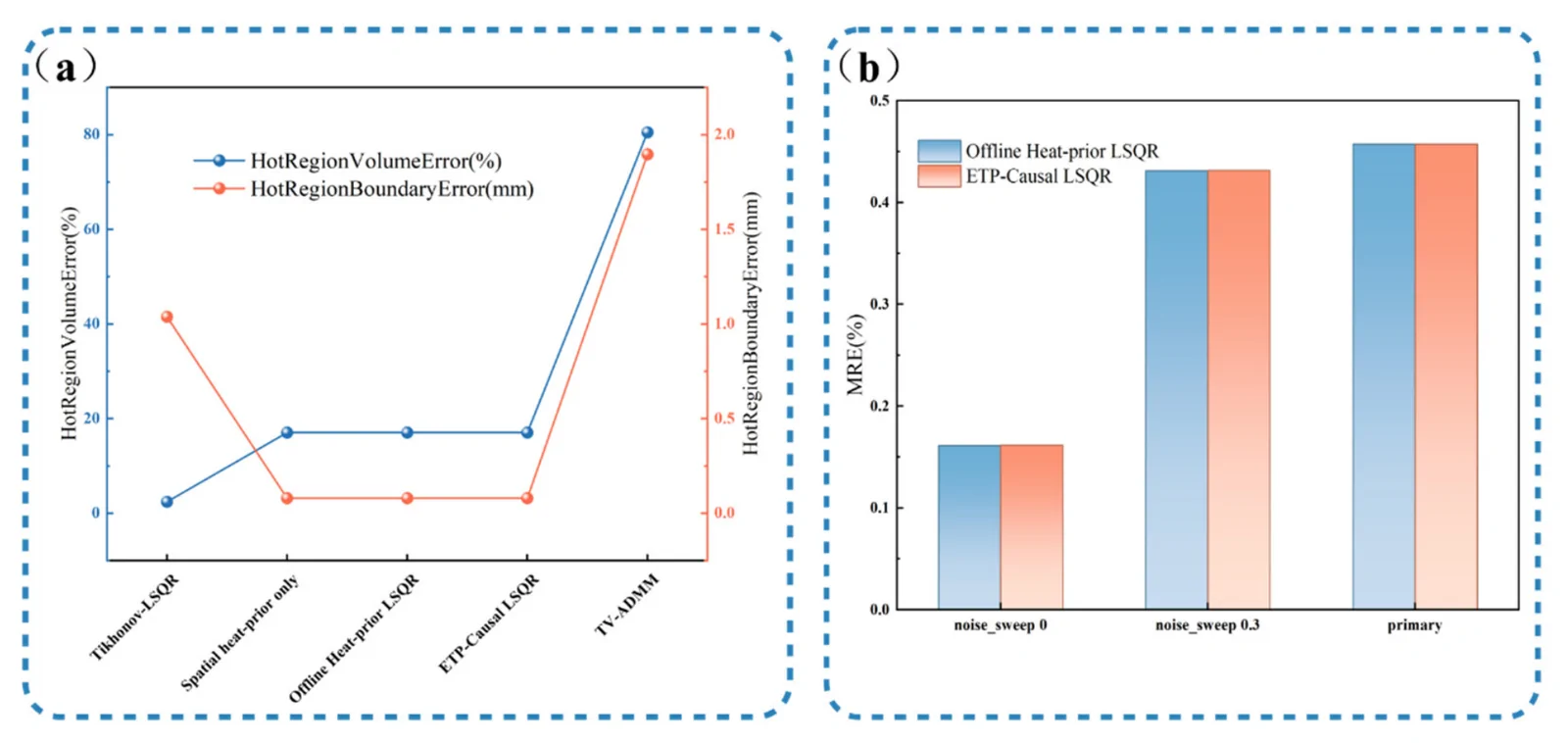
Ablation study metrics. (**a**) Volume and boundary errors of the overheating core region; (**b**) comparison of MRE between the two thermal prior methods under three operating conditions.

Tikhonov-LSQR yields the largest values across all three error metrics, making it the least accurate among all methods. It indicates that relying solely on smoothness regularization proves insufficient to alleviate the ill-posedness in the infrared thermometry inverse problem. Moreover, its Dice and IoU coefficients deviate substantially from the ideal value of 1, indicating inadequate localization capability of the high-temperature core region. Upon introducing the spatial thermal prior, the MRE drops dramatically to 0.467% (Table 4), with overall prediction accuracy markedly improved and reconstruction quality remaining stable across all temperature ranges. The Dice and IoU coefficients both approach unity, demonstrating a significant enhancement in fitting the high-temperature region and directly confirming the substantial role of physical prior constraints in solving the inverse problem. The offline full-space-time thermal prior method, which performs global optimization using temperature data from all time instants, further reduces the MRE to 0.457% (see Table 4), with zero error at the boundary of the overheating core region, representing the performance upper bound for this algorithmic framework. The proposed ETP-Causal LSQR achieves metrics virtually identical to the offline upper bound across all indicators, exhibiting excellent spatial morphology reconstruction capability, with the overheating core region contour more faithfully matching the true state. The TV-ADMM algorithm yields a relatively large average relative error, second only to that of Tikhonov-LSQR. Additionally, its Dice and IoU coefficients severely deviate from the ideal value, resulting in a volume error of the overheating core region more than four times that of the thermal prior methods (Figure 7a). This indicates that TV-ADMM fails to accurately estimate the size of the overheating region or recover the heat source morphology, with reconstruction accuracy significantly inferior to the aforementioned thermal prior algorithms. These data collectively demonstrate that the heat conduction prior algorithms not only exhibit distinct advantages in global error evaluation but also achieve substantial improvements in local hot-spot localization accuracy. To further compare the reconstruction accuracy between the online causal method and the offline upper bound, the MREs of both methods are measured under the ideal noise-free condition, the 0.3% noise condition and the primary operating condition (Figure 7b). Across all three conditions, the errors of the online method are essentially on par with those of the offline method, with the relative error increase not exceeding 1%. This result fully validates the effectiveness of the proposed ETP-Causal LSQR method.

To intuitively quantify the reconstruction performance differences among the various algorithms, this experiment provides visual reconstructions of the temperature fields (Figure 6a–d) and the corresponding absolute error distributions (Figure 6e–h) for auxiliary analysis. The reconstructions from the conventional Tikhonov-LSQR algorithm exhibit pronounced non-physical artifacts: the background temperature distribution is disordered and fluctuates severely, while the boundary of the high-temperature hot-spot core region is blurred and morphologically distorted (Figure 6a). The TV-ADMM reconstructions similarly present staircase-like artifacts with discontinuous boundary transitions (Figure 6b). The reconstructions from offline heat prior and ETP-Causal are virtually identical in visual appearance, with hot-spot contours and temperature gradient transitions exhibiting greater physical consistency, and residual artifacts in the edge regions being further suppressed (Figure 6c,d). In terms of absolute error distribution, the Tikhonov-LSQR algorithm exhibits the highest error magnitude, with global absolute errors generally exceeding 4 K and local maximum errors reaching up to 18 K (Figure 6e), while the other three algorithms show considerably lower error amplitudes. Further, the reconstruction error of Tikhonov-LSQR is spatially diffuse (Figure 6e), which is inherently determined by its L2 spatial smoothing regularization. L2 regularization tends to distribute the error energy uniformly across the entire computational domain, yielding a pattern that is globally smooth yet locally biased everywhere. Moreover, the regularization strength is applied uniformly to all voxels and does not adapt to the local temperature gradient. In deep regions where the signal is severely attenuated, the local error becomes amplified and cannot be suppressed by physical constraints, which is the fundamental reason for the unstable performance of Tikhonov-LSQR in deep-region inversion [23]. The error of TV-ADMM is concentrated near the boundaries of the hot zone and is accompanied by a staircase-like distribution (Figure 6h), which arises from the sparsity-promoting effect of its L1 regularization. During the iterative solution, ADMM forces gradients below a threshold to zero, truncating the continuous diffusive gradients of the true physical field This phenomenon can be attributed to the fact that TV regularization is better suited to piecewise-constant signals, such as sharp edges in image deblurring, whereas for a continuously smooth field like the temperature distribution it creates a structural mismatch [43].

In contrast, methods that incorporate heat conduction priors exhibit localized error distributions, with errors concentrated primarily in the hotspot core rather than being globally diffuse or exhibiting boundary staircasing. The physical origin of this behavior is that the heat conduction prior essentially acts as a constraint on the search space rather than a penalty on gradient magnitude; it does not directly limit the spatial rate of change of the solution, but instead restricts the solution to a subspace compatible with the heat diffusion equation through physical constraints. Consequently, even when deviations remain near the hotspot peak, the reconstructed spatial morphology still retains physical plausibility.

### 3.3. Temporal Temperature Tracking and Overheating Warning Performance

**Figure 8:**
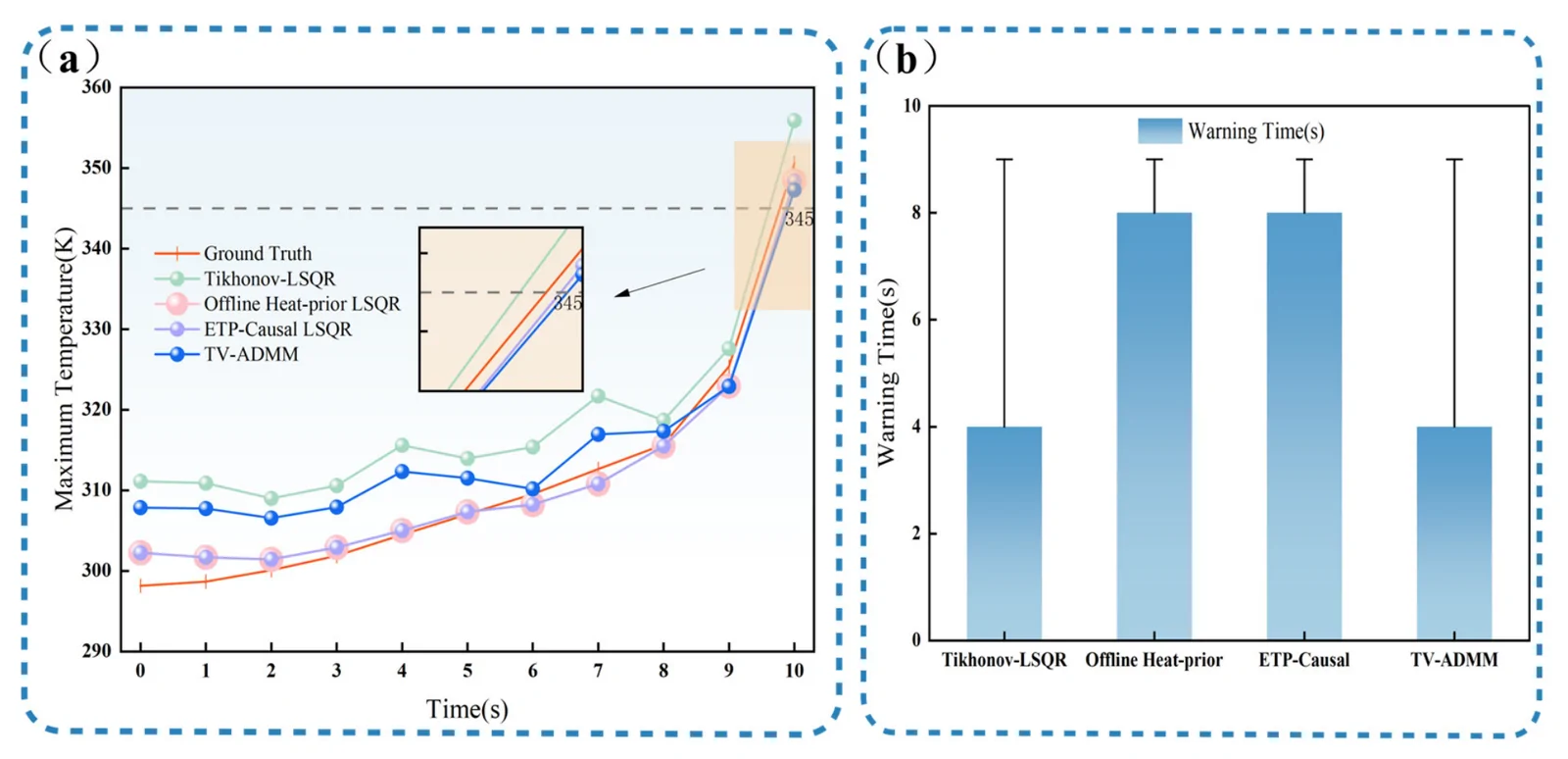
Overheating warning performance. (**a**) evolution of the maximum temperature as a function of energization time; (**b**) comparison of overheating warning times among various algorithms.

To quantitatively evaluate the overheating warning performance of the proposed method, this section assesses the algorithm from two perspectives: temporal temperature tracking accuracy and warning time fidelity. Figure 8a presents the evolution of the maximum temperature over the entire heating process as a function of energization time. The ground-truth curve exhibits a smooth monotonic upward trend, with the heating rate gradually accelerating over time. At t=10s, the peak temperature exceeds 350 K, surpassing the overheating warning threshold (345 K). The Tikhonov-LSQR algorithm exhibits the poorest temporal tracking performance: its reconstructed temperatures are significantly overestimated across the entire time horizon, accompanied by pronounced non-physical fluctuations, with a particularly anomalous drop in the temperature trajectory during the 7~8 s. The TV-ADMM algorithm similarly overestimates the peak temperature during the early heating stage, and its reconstructed temporal trajectory exhibits even more severe oscillations, indicating inferior overall stability. In contrast, the two algorithms incorporating the heat conduction physical prior yield nearly coincident curves that align closely with the ground truth, with minimal maximum temperature deviation throughout the entire heating process. Their temporal tracking smoothness and accuracy are markedly superior to those of the comparison algorithms.

Figure 8b further compares the overheating warning times of the various algorithms. The ground-truth warning time is 9 s, corresponding to the instant when the maximum temperature first reaches the overheating threshold (345 K). To facilitate a consistent evaluation across methods, we define the signed warning time deviation as $\Delta t_{w}=t_{\mathrm{pred}}-t_{\mathrm{true}}$, where negative values indicate early warnings and positive values indicate delayed warnings. The Tikhonov-LSQR and TV-ADMM algorithms both issue warnings at 4 s, yielding $\Delta t_{w}=-5$ s. The offline heat prior and the proposed ETP-Causal LSQR algorithms both issue warnings at 8 s, yielding $\Delta t_{w}=-1$ s. It should be noted that a deviation of −1 s is the minimum non-zero offset detectable at the current temporal resolution of Δt=1 s. Therefore, this value should not be interpreted as indicating a “high-precision” warning; rather, it reflects a one-frame shift intrinsic to the discrete-time simulation framework. Both the −5 s and −1 s deviations indicate early warnings, and the difference between them reflects the relative performance of the reconstruction methods in tracking the temporal evolution of the temperature field. The proposed method consistently yields a smaller absolute deviation, indicating better temporal tracking capability compared with the conventional methods, while the actual significance of the remaining −1 s deviation should be interpreted with respect to the discrete-time resolution of the simulation.

### 3.4. Noise Robustness Analysis and Physical Consistency Evaluation

In practical infrared thermometry environments, measurement noise is inevitable. Owing to the severe ill-posedness of the temperature reconstruction inverse problem based on light-field infrared imaging, slight perturbations in the measured signal can be amplified, leading to significant distortions in the reconstructed temperature field. Therefore, systematically evaluating the robustness of reconstruction algorithms against measurement noise is a critical step in validating the engineering practicality of the proposed method. Following the settings in Section 2, relative noise levels of 0, 0.01, 0.03, 0.05, and 0.07 are prescribed, and temperature reconstructions are performed under each noise level to assess reconstruction accuracy and algorithmic noise robustness.

**Figure 9:**
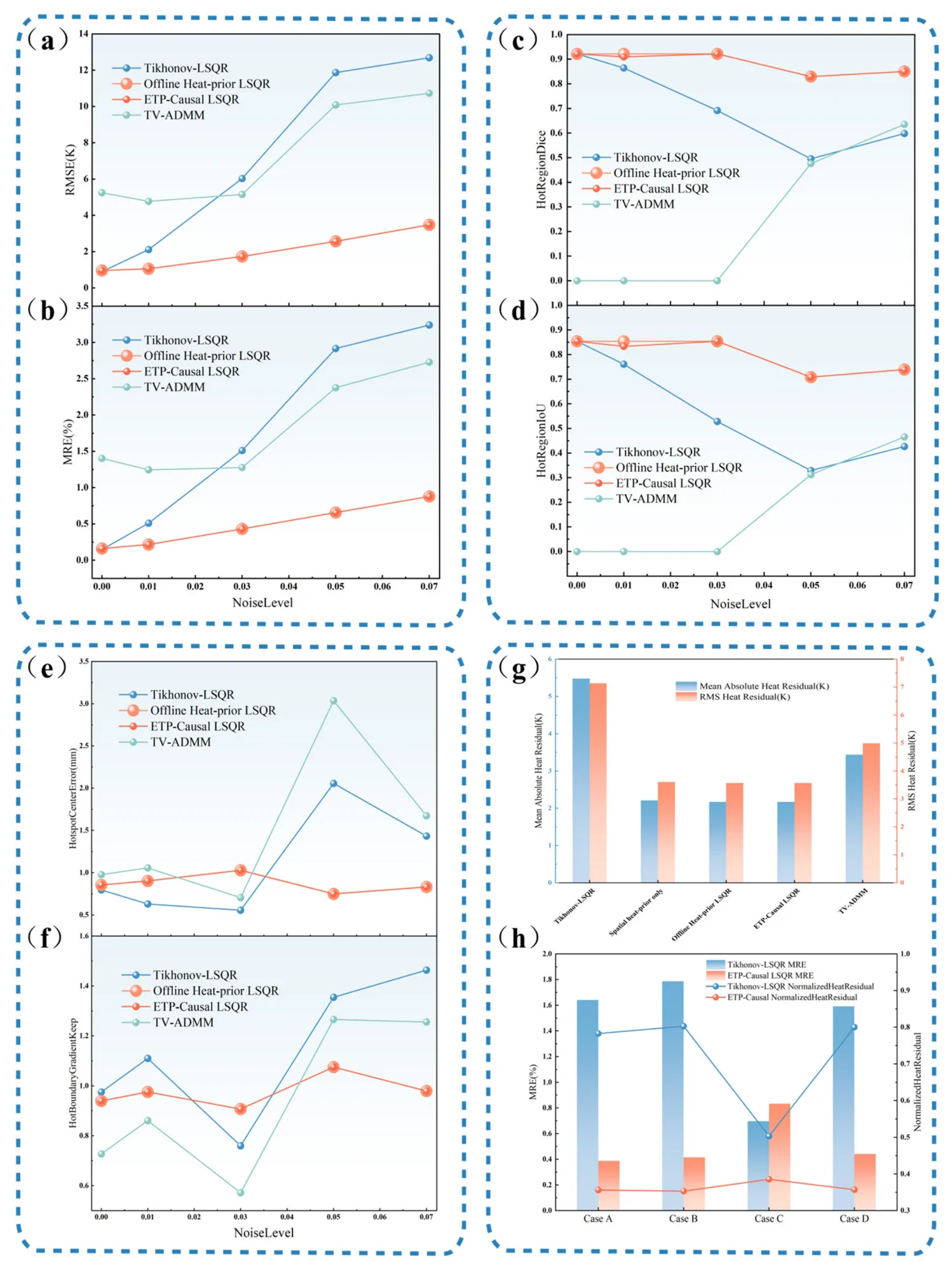
Comprehensive performance evaluation. (**a**) RMSE comparison under different noise levels; (**b**) MRE comparison under different noise levels; (**c**) Dice coefficient comparison of the high-temperature region under different noise levels; (**d**) IoU coefficient comparison of the high-temperature region under different noise levels; (**e**) hot-spot center localization error comparison; (**f**) Comparison of gradient preservation at high-temperature boundaries under different noise levels; (**g**) Comparison of mean absolute thermal residuals and root mean square thermal residuals for different algorithms; (**h**) Comparison of MRE and normalized heat equation residuals for the two types of algorithms under generalized conditions.

As shown in Figure 9a, the conventional Tikhonov-LSQR exhibits a sharp increase in RMSE with increasing noise level, rising from approximately 0.9 K to 12.7 K, indicating that the standard regularized LSQR solver is highly sensitive to measurement perturbations. TV-ADMM exhibits a substantial baseline error (approximately 5.2 K) even under noise-free conditions and severely degrades when the noise level exceeds 0.03, with RMSE exceeding 10 K. In contrast, the proposed ETP-Causal LSQR maintains the lowest RMSE across all noise levels, with a gentle increase from approximately 0.9 K to approximately 3.4 K, and its curve coincides with that of the offline thermal prior method. The MRE curves exhibit a consistent trend (Figure 9b): the MRE of ETP-Causal LSQR remains below 0.9% even at a noise level of 0.07, whereas both Tikhonov-LSQR and TV-ADMM exceed 2.5%. Notably, the error growth rate of ETP-Causal LSQR is approximately linear and significantly lower than that of the comparison methods, indicating that the embedded causal thermal prior effectively constrains the solution space and suppresses noise amplification.

However, global temperature fidelity under noisy conditions does not adequately reflect the spatial localization accuracy of the local hot zone, which is of greater concern in thermal monitoring applications. Figure 9c,d present the Dice and IoU coefficients of the hot zone, quantifying the spatial overlap between the reconstructed and true hot regions. The results reveal pronounced differences among the methods. The Dice coefficient of Tikhonov-LSQR monotonically decreases from 0.92 to 0.59, and the IoU from 0.85 to 0.42, indicating that although its global mean error remains acceptable under moderate noise, the geometric morphology of the hot zone undergoes progressive distortion. More critically, TV-ADMM completely fails to identify the hot zone at low-to-moderate noise levels, with both Dice and IoU approaching zero. This phenomenon can be attributed to the aggressive piecewise-constant smoothing effect induced by total variation regularization: when the signal is perturbed, TV regularization excessively suppresses hot-zone contrast, effectively “flattening” the hot region. However, TV-ADMM exhibits partial recovery at noise levels of 0.05 and 0.07, presumably because strong noise disrupts the TV sparsity assumption, causing the solver to revert to a solution with smaller bias. In stark contrast, the proposed ETP-Causal LSQR maintains Dice coefficients consistently above 0.82 and IoU above 0.70 across the entire noise range, with nearly flat curves that coincide with those of the offline method. This result demonstrates that the causal thermal prior not only stabilizes temperature amplitude reconstruction but also preserves the spatial structure of the hot zone.

Figure 9e,f further evaluate the spatial stability of the reconstructed hot spot. As shown in Figure 9e, the hot-spot center error of ETP-Causal LSQR remains highly stable across the entire noise range, fluctuating between 0.74 and 1.02 mm, significantly lower than that of Tikhonov-LSQR (reaching 2.05 mm at a noise level of 0.05) and TV-ADMM (reaching 3.03 mm at a noise level of 0.05). The superior center localization accuracy of the proposed method is attributed to the causal temporal coherence constraint, which effectively prevents hot-spot drift under perturbations. In terms of boundary fidelity, Figure 9f shows that the thermal boundary gradient preservation of ETP-Causal LSQR remains consistently close to the ideal value of 1, indicating that the temperature gradient at the hot-zone boundary is well maintained. In contrast, TV-ADMM exhibits a pronounced valley at a noise level of 0.03, which corresponds precisely to its complete misidentification of the hot zone in Figure 9e. Collectively, these results demonstrate that ETP-Causal LSQR achieves an optimal balance between noise suppression and detail preservation; TV-ADMM tends to oversmooth the flame structure, while Tikhonov-LSQR suffers from noise-induced blurring artifacts.

The superior noise robustness of ETP-Causal LSQR can be attributed to the following factors. First, the ETP-Causal framework imposes a dynamically updated causal prior derived from the physical heat transfer equation, which adaptively adjusts the regularization strength according to the local signal-to-noise ratio, thereby avoiding the under-regularization of Tikhonov-LSQR and the over-regularization of TV-ADMM. Second, by exploiting the temporal causality of thermal diffusion, the method effectively discriminates between coherent hot-spot infrared signals and incoherent measurement noise in the time domain, which is particularly advantageous for continuous monitoring scenarios where adjacent frames are physically correlated. Therefore, the proposed ETP-Causal LSQR method exhibits strong robustness against measurement noise and is well suited for temperature measurement of flexible heating patches.

The reliability of temperature field reconstruction depends not only on the fidelity of fitting the measurement data but, more fundamentally, on the degree to which the reconstructed results satisfy the underlying physical governing equations. To this end, this work adopts the mean absolute thermal residual $R_{\mathrm{MA}}$ and the root mean square thermal residual $R_{\mathrm{RMS}}$ as quantitative metrics for physical consistency, where smaller residuals indicate that the reconstructed field more strictly adheres to the physical laws of energy conservation and thermal diffusion.

As shown in Figure 9g, the physical consistency of the various methods exhibits distinct stratification. The RMS thermal residual of Tikhonov-LSQR reaches as high as 7.1 K, indicating that although its reconstruction achieves mathematical smoothness at the pixel level, it substantially deviates from the heat conduction governing equation and lacks physical interpretability. The residual of TV-ADMM is 5 K, also exhibiting poor physical consistency, suggesting that although total variation regularization suppresses noise, it does not incorporate genuine heat transfer physics and thus cannot guarantee that the reconstructed field satisfies energy conservation.

Upon introducing the spatial thermal prior (offline heat prior LSQR), the RMS thermal residual decreases to 3.6 K, representing a 28% reduction relative to TV-ADMM. This significant improvement validates the strong constraining effect of the physical prior on the solution space: when the regularization term is replaced from a purely mathematical norm to a physical residual derived from the heat conduction equation, the reconstruction process is guided toward the solution neighborhood that conforms to the true heat transfer physics. In comparison, the offline full-space-time thermal prior and the proposed ETP-Causal LSQR achieved residuals of 3.5 K and a comparable level, respectively, attaining the optimal values among all methods (Figure 9g).

The above experiments are all conducted under the primary operating condition. Furthermore, when facing unseen operating conditions with variations in heating power, boundary conditions or heat dissipation characteristics, models lacking physical interpretability often exhibit catastrophic performance degradation. To validate this conjecture, this work further conducts generalization experiments across operating conditions, with four generalization cases designed: Case A (center hotspot), Case B (electrode edge hotspot), Case C (dual offset hotspots), and Case D (stronger surface cooling). The fixed parameters calibrated under the primary operating condition are directly transferred for testing to evaluate the reconstruction and warning performance of each model in out-of-distribution scenarios. To demonstrate the physical consistency of each algorithm under these generalization cases, the normalized thermal residual $R_{N}$ is adopted as the evaluation metric.

Figure 9h presents a comparison of reconstruction accuracy and physical consistency between Tikhonov-LSQR and ETP-Causal LSQR across the four generalization cases. The results reveal significant performance divergence between the two methods in unseen scenarios outside the training domain: Tikhonov-LSQR exhibits MRE exceeding 1.5% in Cases A, B, and D, indicating pronounced generalization degradation; by contrast, ETP-Causal LSQR maintains MRE below 0.85% across all cases, with the normalized thermal residual consistently stabilized at a low level of 0.35–0.39, verifying the enhancement of cross-operating condition generalization capability conferred by the causal heat transfer prior.

Specifically, under the three cases of center hot-spot migration (Case A), electrode-edge hot-spot (Case B), and enhanced surface cooling (Case D), the MREs of ETP-Causal LSQR are 0.38%, 0.41%, and 0.44%, respectively, representing approximately 75% reductions compared with Tikhonov-LSQR (1.65%, 1.78%, and 1.59%). This significant advantage originates from the strong constraint of the embedded physical prior on the solution space: when boundary conditions or heat source locations change, the causal heat conduction equation still correctly characterizes the spatiotemporal evolution trend of the temperature field, ensuring that the reconstruction remains within the physically feasible domain.

It is noteworthy that in the dual-offset hot-spot case (Case C), the MRE of ETP-Causal LSQR (0.83%) is slightly higher than that of Tikhonov-LSQR (0.70%). This phenomenon can be attributed to the fact that the temperature field in this case exhibits a bimodal asymmetric distribution, which presents a structural discrepancy from the single-hot-spot Gaussian thermal prior calibrated under the primary operating condition, thereby limiting the fitting flexibility of the model to complex thermal distributions to a certain extent. Nevertheless, even in this most unfavorable case, the MRE of ETP-Causal LSQR remains below 1%, satisfying engineering accuracy requirements.

From the perspective of physical consistency, the normalized thermal residual of ETP-Causal LSQR remains nearly invariant across the four cases (0.35–0.39), with a fluctuation amplitude of less than 20%, demonstrating strong robustness against operating condition variations. In contrast, the residual of Tikhonov-LSQR reaches as high as 0.78–0.80 in Cases A, B, and D, and drops to 0.50 in Case C, exhibiting fluctuation characteristics that are not fully coupled with the MRE trend. This suggests that the reconstruction errors of Tikhonov-LSQR carry a risk of spurious accuracy. The error may occasionally be low in certain cases, but the reconstructed field has already severely deviated from the thermal diffusion physics in essence.

### 3.5. Computational Efficiency and Iterative Characteristics Analysis

**Figure 10:**
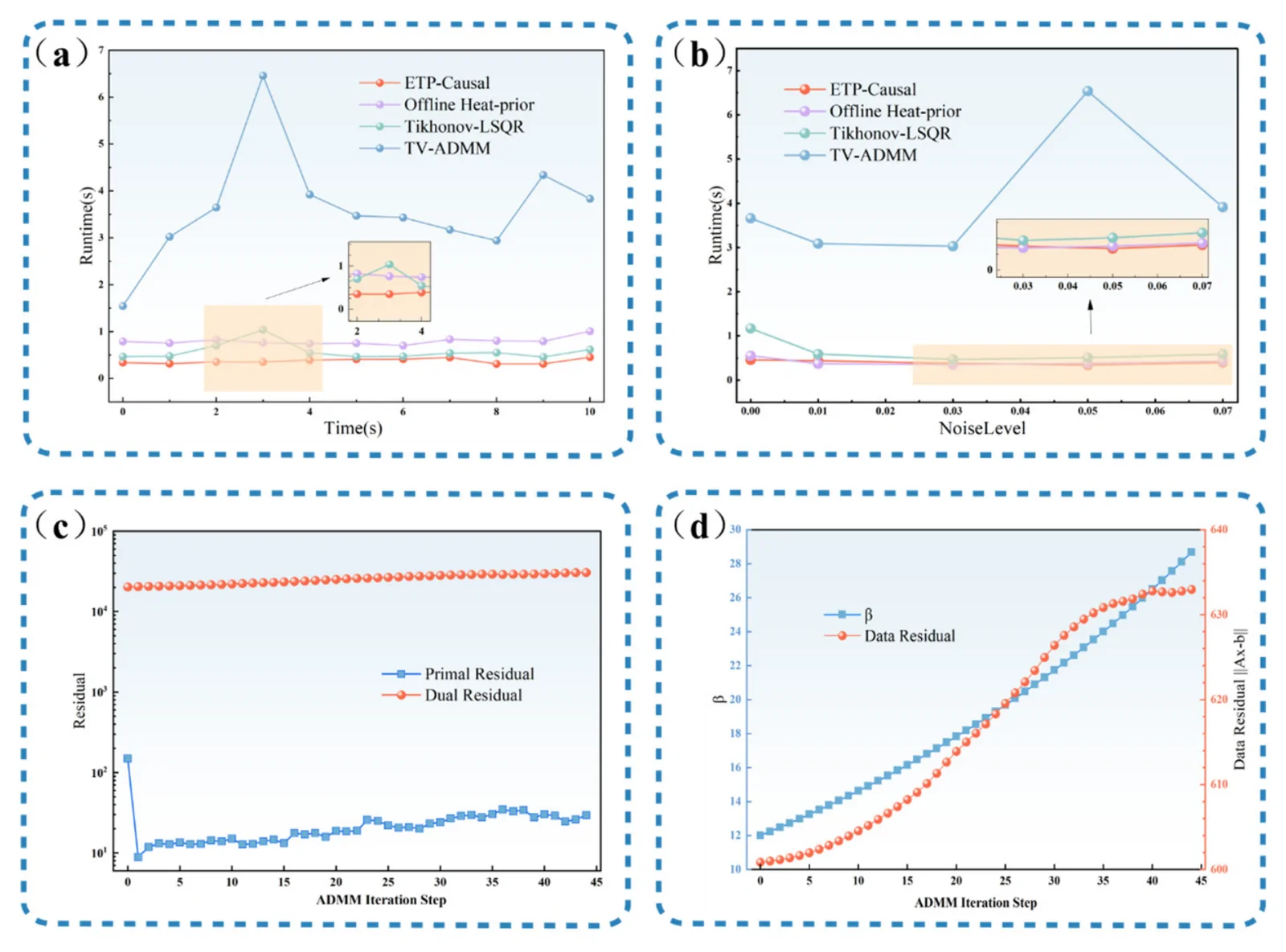
Computational efficiency of various algorithms. (**a**) Cumulative runtime during the 0–10  s heating process under the primary operating condition; (**b**) cumulative runtime at t=10 s under different noise levels; (**c**) iteration residual curves of TV-ADMM; (**d**) data residuals of TV-ADMM under the parameter continuation strategy.

Online real-time monitoring imposes strict requirements on the computational efficiency of the algorithm. Figure 10a,b present the cumulative runtime comparisons of the various algorithms. As shown in Figure 10a, over the entire 0–10 s heating process, the cumulative runtime of ETP-Causal LSQR remains consistently stable within the range of 0.31–0.45 s (mean 0.37 s), with minimal temporal fluctuation, demonstrating excellent computational stability. Tikhonov-LSQR and offline heat prior exhibit cumulative runtimes of approximately 0.58 s and 0.80 s, respectively, with no significant temporal drift, yet their absolute runtimes are both higher than that of ETP-Causal. In contrast, TV-ADMM exhibits pronounced temporally non-stationary computational overhead: its cumulative runtime surges sharply to 6.46 s at the early heating stage and subsequently declines to the 3–4 s range, failing to guarantee a consistent real-time response. Figure 10b further examines the influence of measurement noise on computational overhead. The cumulative runtime of ETP-Causal LSQR remains nearly unchanged with increasing noise levels, indicating that the embedded causal heat transfer prior provides a high-quality physical initial guess for the LSQR iterations, rendering the convergence process insensitive to data perturbations. Tikhonov-LSQR and offline heat prior similarly exhibit favorable noise robustness. However, TV-ADMM exhibits a pronounced runtime peak at a noise level of 0.05 (6.54 s), representing an approximately 78% increase compared with its noise-free case, indicating that the iterative solution of total variation regularization falls into suboptimal paths under specific noise conditions, further exposing its computational reliability concerns.

This work further compares the average per-frame runtime of each algorithm (Table 5). ETP-Causal LSQR requires only 0.079 s per frame, representing a reduction of approximately 24% compared with Tikhonov-LSQR, approximately 50% compared with offline heat prior, and approximately 85% compared with TV-ADMM. This advantage stems from the fact that the ETP-Causal framework uses the reconstruction result from the previous time step as the physical initial guess for the LSQR iterations at the current step, effectively compressing the number of iterations required, rather than relying on time-consuming adaptive searches for regularization parameters. For typical light-field infrared imaging systems (with frame rates typically exceeding 10 Hz), the per-frame runtime of ETP-Causal LSQR is well below the 100 ms real-time threshold, fully satisfying the timing requirements for online reconstruction. By contrast, the average runtime of TV-ADMM exceeds 500 ms, which cannot keep pace with the sampling rate and thus lacks engineering feasibility for real-time monitoring.

To further elucidate the root cause of TV-ADMM’s low efficiency, Figure 10c presents the iteration residual curves of TV-ADMM. The primal residual decreases rapidly during the early iterations, then oscillates persistently at a low level without converging to a sufficiently small value. Meanwhile, the dual residual increases monotonically throughout the process, remaining at a high magnitude with no sign of convergence, indicating poor convergence behavior of ADMM for this inverse problem. Figure 10d shows the residual variation under the parameter continuation strategy. As the regularization parameter is gradually increased, the data residual rises correspondingly and continuously, without any plateau or downward trend throughout the process. This suggests that as the TV regularization constraint intensifies, the algorithm continues to sacrifice data fitting accuracy, resulting in a solution that deviates substantially from the measurement data, and fails to achieve a balance between data fidelity and regularization constraints.

## 4. Conclusions

In this work, taking PEDOT:PSS conductive polymer gel flexible heating patches as the research object and targeting the localized overheating risks induced by spatially nonuniform electrical conductivity in flexible electronic devices, we propose a three-dimensional temperature field inversion and overheating warning method based on focused light-field infrared imaging. The proposed method establishes a complete physically constrained framework encompassing forward electro-thermal coupled temperature field simulation, forward focused light-field infrared imaging modeling, and causal online inversion, thereby enabling real-time monitoring of the internal three-dimensional temperature field without compromising device structural integrity. Through systematic numerical simulation experiments, the performance of the proposed method is comprehensively validated in terms of reconstruction accuracy, noise robustness, physical consistency, generalization capability, and computational efficiency. The main conclusions are summarized as follows:

(1) In terms of three-dimensional temperature field reconstruction accuracy, the ETP-Causal LSQR achieves an MRE of 0.457% under the primary operating condition (noise level 0.03), representing a 71.84% reduction compared with the conventional Tikhonov-LSQR (1.623%), and performs on par with the performance upper bound of offline global optimization. This result demonstrates that by transforming historical reconstruction information into a physical prior for the current time step via the heat conduction equation and incorporating it as a soft constraint into the inversion process, the proposed method attains reconstruction performance comparable to offline optimization while strictly satisfying the causality requirement.
(2) In terms of overheating warning reliability, the proposed method achieves temporal tracking deviation of the maximum temperature within 1 K, and the warning time deviates from the ground truth by only 1 s, effectively avoiding the premature false alarms exhibited by Tikhonov-LSQR and TV-ADMM.
(3) In terms of noise robustness, across the noise range from 0 to 0.07, the ETP-Causal LSQR maintains consistently low mean relative errors, with the hot-spot localization error stabilized within 1 mm, exhibiting far less performance degradation than Tikhonov-LSQR and TV-ADMM. The heat conduction prior effectively anchors the diffusion center and spatial morphology of the hot spot from physical principles, enabling the reconstruction to maintain high reliability even under severe noise conditions.
(4) In terms of physical consistency, the ETP-Causal LSQR reconstruction strictly adheres to the heat conduction law and exhibits favorable physical interpretability. In terms of generalization capability and computational efficiency, when the fixed parameters are transferred across four generalization cases, the MRE of ETP-Causal LSQR remains stable between 0.388% and 0.438%, eliminating the need for repeated parameter recalibration for new operating conditions. The single-frame reconstruction requires only 0.09 s, outperforming the other algorithms and satisfying the online real-time monitoring requirements of infrared thermometry systems.

Nevertheless, this study has certain limitations. The current validation is based on numerical simulations, and experimental measurements of the internal temperature field in real soft-material devices have not yet been performed. It is worth noting, however, that light-field imaging has already been extensively validated experimentally in combustion diagnostics. A focused light-field camera was employed to reconstruct the three-dimensional temperature field of an ethylene diffusion flame, achieving a deviation of less than 6.7% compared with high-precision thermocouple measurements [44]. It demonstrates that light-field imaging can achieve reconstruction accuracy comparable to that of thermocouples in practical combustion diagnostics, confirming the feasibility of translating such methods from simulation to experiment.

The simulation accuracy of the present work under the synthetic model (MRE=0.457% for the primary case) is superior to the experimentally reported accuracies cited above. This not only reflects the performance potential of the proposed ETP-Causal LSQR algorithm under idealized conditions but also implies that, when extended to real PEDOT:PSS gel experiments, the accumulated effects of material parameter uncertainties, emissivity and optical calibration errors, and contact thermal resistance will lead to reconstruction accuracy lower than the numerical results reported here. Nevertheless, considering the measured accuracies of the aforementioned light-field thermometry experiment (deviation<6.7%) and the noise robustness demonstrated by the present method in the sensitivity analysis, the reconstruction error in practical experiments is expected to remain within an engineering-acceptable range. Accordingly, this work positions itself at the stage of numerical framework validation; subsequent efforts should establish a physical experimental platform, perform independent thermal property characterization for specific gel formulations, and carry out radiometric calibration and embedded-thermocouple comparison experiments to reliably assess the actual accuracy and recalibrate the heat source strength coefficient. Furthermore, for scenarios involving nonlinear thermal effects such as phase change or temperature-dependent thermal conductivity, the accuracy of the linear heat conduction prior may degrade; future work may incorporate nonlinear heat conduction models to extend the applicability of the method.

## Figures and Tables

**Table 1:** Parameter settings of the focused light-field camera.

Parameters	Value
Aperture radius (main lens) (mm)	3.568
Diameter of micro lens (mm)	0.165
Focal length of the main lens (mm)	50
Focal length of the micro lens (mm)	0.8
Distance between ML and patch(mm)	750
Distance between ML and MLA (mm)	55
Distance between MLA and CCD (mm)	1
Microlens shape	Circle
Pixel resolution of CCD	4000 × 4000
Diameter of pixel (mm)	0.0075
Number of micro lens	18 × 16

**Table 2:** Optimal regularization parameters of the ETP Causal LSQR algorithm.

Parameter	Optimal Value
λ	3.162
μ	0.001

**Table 3:** Reconstruction accuracy metrics of the heat prior method under the noise-free scenario.

Method	RMSE	MAE	MRE	Dice	IoU
Offline heat prior LSQR	0.964	0.526	0.161	0.921	0.854
ETP-Causal LSQR	0.964	0.526	0.161	0.921	0.854

**Table 4:** Comprehensive performance comparison of various algorithms at t=10 s under the primary operating condition.

Method	RMSE	MAE	MRE	Dice	IoU
Tikhonov-LSQR	6.498	5.130	1.623	0.543	0.373
Spatial heat prior only	1.865	1.482	0.467	0.921	0.854
Offline heat prior LSQR	1.837	1.451	0.457	0.921	0.854
ETP-Causal LSQR	1.837	1.451	0.457	0.921	0.854
TV-ADMM	5.477	4.177	1.314	0.327	0.195

**Table 5:** Average per-frame computational time of the four methods.

Method	Average Runtime (s)
Tikhonov-LSQR	0.104
Offline Heat prior	0.157
ETP-Causal	0.079
TV-ADMM	0.543

## Data Availability

The original contributions presented in this study are included in the article. Further inquiries can be directed to the corresponding author.

## Abbreviations

The following abbreviations are used in this manuscript:

ETP	Electro-Thermal Physics
LSQR	Least Squares Quasi-orthogonal matrix Right-triangle matrix
QR	Quasi-orthogonal matrix Right-triangle matrix
TV-ADMM	Total Variation with Alternating Direction Method of Multipliers
PEDOT:PSS	Poly(3,4-ethylenedioxythiophene):polystyrene sulfonate
